# The gut microbiota contributes to the pathogenesis of anorexia nervosa in humans and mice

**DOI:** 10.1038/s41564-023-01355-5

**Published:** 2023-04-17

**Authors:** Yong Fan, René Klinkby Støving, Samar Berreira Ibraim, Tuulia Hyötyläinen, Florence Thirion, Tulika Arora, Liwei Lyu, Evelina Stankevic, Tue Haldor Hansen, Pierre Déchelotte, Tim Sinioja, Oddny Ragnarsdottir, Nicolas Pons, Nathalie Galleron, Benoît Quinquis, Florence Levenez, Hugo Roume, Gwen Falony, Sara Vieira-Silva, Jeroen Raes, Loa Clausen, Gry Kjaersdam Telléus, Fredrik Bäckhed, Matej Oresic, S. Dusko Ehrlich, Oluf Pedersen

**Affiliations:** 1 Novo Nordisk Foundation Center for Basic Metabolic Research, Faculty of Health and Medical Science, University of Copenhagen, Copenhagen, Denmark; 2 Center for Eating Disorders, Odense University Hospital, and Research Unit for Medical Endocrinology, Mental Health Services in the Region of Southern Denmark, Open Patient data Explorative Network (OPEN) and Clinical Institute, University of Southern Denmark, Odense, Denmark; 3 Université Paris-Saclay, INRAE, MGP, Jouy-en-Josas, France; 4 School of Science and Technology, Örebro University, Örebro, Sweden; 5 Department of Medicine, University of Copenhagen and Herlev-Gentofte University Hospital, Copenhagen, Denmark; 6 INSERM U1073 Research Unit and TargEDys, Rouen University, Rouen, France; 7 Laboratory of Molecular bacteriology, Department of Microbiology and Immunology, Rega Institute Ku Leuven, Leuven, Belgium; 8 Center for Microbiology, VIB, Leuven, Belgium; 9 Institute of Medical Microbiology and Hygiene and Research Center for Immunotherapy (FZI), University Medical Center of the Johannes Gutenberg-University Mainz, Mainz, Germany; 10 Institute of Molecular Biology (IMB), Mainz, Germany; 11 Department of Child and Adolescent Psychiatry, Aarhus University Hospital, Aarhus, Denmark; 12 Department of Clinical Medicine, Faculty of Health, Aarhus University, Aarhus, Denmark; 13 Unit for Psychiatric Research, Aalborg University Hospital, Aalborg, Denmark; 14 Department of Communication and Psychology, The Faculty of Social Sciences and Humanities, Aalborg University, Aalborg, Denmark; 15 The Wallenberg Laboratory, Department of Molecular and Clinical Medicine, Institute of Medicine, Sahlgrenska Academy, University of Gothenburg, Gothenburg, Sweden; 16 Department of Clinical Physiology, Sahlgrenska University Hospital, Region Västra Götaland, Gothenburg, Sweden; 17 School of Medical Sciences, Örebro University, Örebro, Sweden; 18 Turku Bioscience Centre, University of Turku and Åbo Akademi University, Turku, Finland; 19 Department of Clinical and Movement Neurosciences, University College London, London, UK

**Keywords:** Neurological disorders, Metabolic disorders, Nutrition disorders

## Abstract

Anorexia nervosa (AN) is an eating disorder with a high mortality. About 95% of cases are women and it has a population prevalence of about 1%, but evidence-based treatment is lacking. The pathogenesis of AN probably involves genetics and various environmental factors, and an altered gut microbiota has been observed in individuals with AN using amplicon sequencing and relatively small cohorts. Here we investigated whether a disrupted gut microbiota contributes to AN pathogenesis. Shotgun metagenomics and metabolomics were performed on faecal and serum samples, respectively, from a cohort of 77 females with AN and 70 healthy females. Multiple bacterial taxa (for example, *Clostridium* species) were altered in AN and correlated with estimates of eating behaviour and mental health. The gut virome was also altered in AN including a reduction in viral–bacterial interactions. Bacterial functional modules associated with the degradation of neurotransmitters were enriched in AN and various structural variants in bacteria were linked to metabolic features of AN. Serum metabolomics revealed an increase in metabolites associated with reduced food intake (for example, indole-3-propionic acid). Causal inference analyses implied that serum bacterial metabolites are potentially mediating the impact of an altered gut microbiota on AN behaviour. Further, we performed faecal microbiota transplantation from AN cases to germ-free mice under energy-restricted feeding to mirror AN eating behaviour. We found that the reduced weight gain and induced hypothalamic and adipose tissue gene expression were related to aberrant energy metabolism and eating behaviour. Our ‘omics’ and mechanistic studies imply that a disruptive gut microbiome may contribute to AN pathogenesis.

## Abstract (web summary)

Faecal metagenomics and serum metabolomics reveal compositional and functional alterations in the gut microbiota of women with anorexia nervosa, and faecal transplants could transfer an anorexia-associated phenotype to germ-free mice.

## Main

Anorexia nervosa (AN) is a serious mental health condition and eating disorder characterized by distorted body image, obsessive thoughts about food, ritualistic patterns of behaviour including reduced food intake, loss of body weight, raised physical activity and emotional rigidity^1^. AN primarily affects women in about 95% of cases and has a population prevalence of about 1%^2^. It can be classified into two subtypes, the common restricting (AN-RS) type and the less prevalent binge-eating or purging (AN-BP) type^1^. The evidence base for treatment is lacking^3^ and although specialized multidisciplinary treatment can reduce mortality^4^, less than half of AN cases achieve complete remission^5^. The aggregate mortality rate is estimated to be 5.6% per decade, much higher than in the general population^6^.

Despite research to determine the aetiology of AN, it remains a syndrome, that is, a collection of symptoms without a well-defined unifying cause. Twin studies have reported heritability estimates of 50–60%^7^ and genome-wide association studies have identified eight genomic loci showing correlations with psychiatric disorders, physical activity, and metabolic and anthropometric traits. This is independent of common variants associated with body-mass index^8,9^. At the pathophysiological level, AN is characterized by multiple endocrine changes^10^ and perturbed signalling of neurotransmitters in various parts of the brain^11^.

The human digestive tract contains complex assemblies of microorganisms that can impact host metabolism, immunity and neurobiology via metabolites and other pathways^12^. This may include the gut-microbiota-brain axis, which can affect brain functions including regulation of appetite, behaviour and emotions^13^. For example, the bacterial metabolite caseinolytic peptidase B (ClpB), predominantly produced by enterobacteria, is an antigenic mimic of α-melanocyte-stimulating hormone, which can exert anorexigenic effects^14,15^.

It has been hypothesized that an aberrant gut microbiota may be involved in the pathogenesis of AN. Several small studies that used amplicon sequencing to characterize the gut microbiota at the genus level in AN have been published^16–19^, showing dysbiosis of gut bacterial microbiota (see Supplementary Note 1). Moreover, in a mouse model of anorexia, changes in the gut microbiota have been shown to be associated with changes in eating behaviour and expression of hypothalamic neuropeptides^20^.

**Extended Data Fig. 1:**
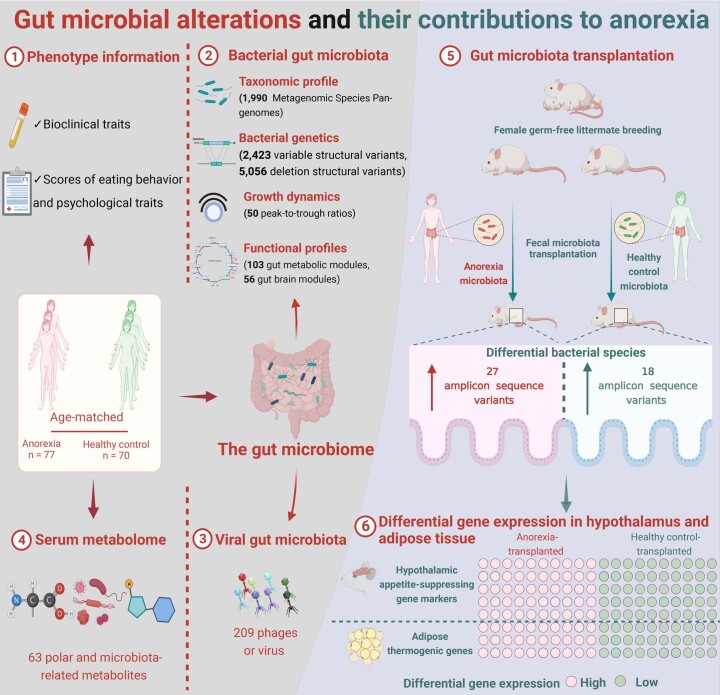
Graphical abstract of the study workflow and findings. Workflow was created with BioRender.com.

Here we explored the hypothesis that a perturbed intestinal gut microbiota and serum metabolome contribute to the complex pathogenesis of AN. To do so, we performed shotgun metagenomics on faecal samples from 77 female AN cases and 70 age-matched female controls allowing for in-depth analyses of the gut bacterial and archaeal microbiota at taxonomic, functional and genetics levels, as well as analyses of the viral gut microbiota. We also characterized the serum metabolome, which was analysed with the gut metagenome data in relation to individual markers of eating- and psychological behaviour. Causal mechanisms were explored in silico using bidirectional mediation analyses and in vivo through faecal microbiota transplantation (FMT) of gut microbiota from AN cases to female germ-free littermates. Our findings lend support to the hypothesis that a disrupted AN gut microbiota and associated bacterial metabolites contribute to AN pathogenesis (Extended Data Fig. 1).

## Results

### Phenotypes of women with AN and controls

Summarized statistics of clinical characteristics of the 77 enrolled women with AN and 70 age-matched control women considered to be of healthy weight (HC) are given in Supplementary Table 1. The validated Eating Disorder Inventory-3 (EDI-3) questionnaire was used to estimate levels of specific eating behaviour^21^ and a detailed description of the EDI-3 subscale is shown in Supplementary Note 2. As expected, women with AN were much leaner, had lower fasting serum concentrations of glucose and insulin, higher insulin sensitivity as estimated by homoeostatic model assessment for insulin resistance (HOMA-IR) and lower serum C-reactive protein. Detailed baseline characteristics of study participants are shown in Supplementary Table 2. Additionally, within AN cases, AN-RS type cases were characterized by higher values of serum insulin and lower insulin sensitivity than AN-BP individuals (Supplementary Table 1). Comparing stool samples from AN and HC, there was no significant difference in bacterial cell counts between AN and HC or within subtypes of AN (*P*_Wilcoxon_ > 0.05, Supplementary Table 1).

### Gut microbiota composition is altered in AN

**Extended Data Fig. 2:**
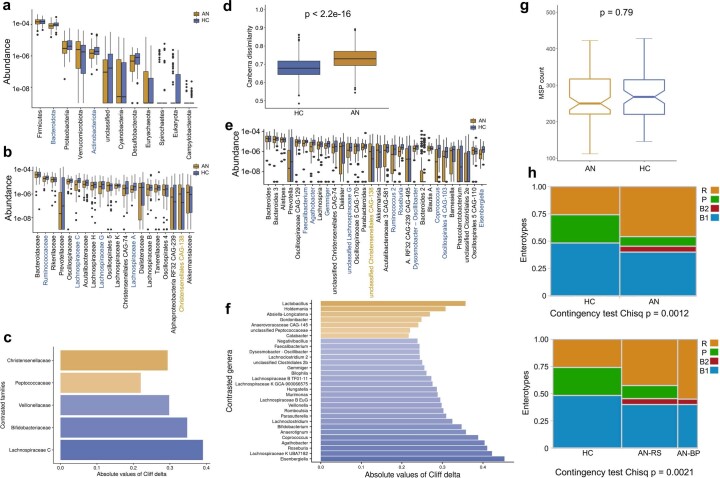
Taxonomic differences at phylum, family and genus level and differences in enterotypes in cases with AN compared with healthy women. **a**,**b**,**e** Box plot (line, median; box, interquartile range (IQR); whiskers, 1.5× IQR) of comparison between AN (gold, *n* = 77) and HC (blue, *n* = 70) of relative abundance of the 12 bacterial phyla detected in at least 10% of individuals (**a**), the 20 most abundant bacterial families (**b**), and the top 30 genera (**e**). Features are sorted by decreasing mean abundance. Zero values are set to 1e-10. Features colored in blue are enriched in HC group, and gold are enriched in AN group. Significance was determined by two-sided Wilcoxon rank-sum test, followed by drug-deconfounding and multiple testing correction by Benjamini-Hochberg method on all features (**a**,**b**,and **e**, see Source Data for exact p values). **c**,**f**, Absolute values of Cliff’s Delta effect size of families (**c**) and genera (**f**) contrasted between AN and HC after drug-deconfounding (adjusted p-value ≤ 0.1). Gold barplots indicate features more abundant in AN; blue barplots indicate features more abundant in HC (**c**,**f**). **d**,**g**, Box plot (line, median; box, IQR; whiskers, 1.5× IQR) showing β-diversity (Canberra distance) of gut bacteriome at genus level (**d**) and richness of Metagenomic Species Pan-genomes (MSPs) (**g**) between AN (gold, *n* = 77) and HC (blue, *n* = 70). Significance was determined by two-sided Wilcoxon rank-sum test (**d**,**g**). **h**, Upper panel demonstrates enterotype prevalence in AN (*n* = 77) and HC (*n* = 70), lower panel shows enterotype prevalence in HC and AN patients split into AN-RS (*n* = 56) and AN-BP (*n* = 21) groups. Source data

At the phylum level, AN microbiota samples were characterized by a reduction in Bacteroidota and Actinobacteriota (Extended Data Fig. 2a). At the family level, Bacteroidaceae was dominant in both groups (Extended Data Fig. 2b). Among the top 20 most abundant families, the abundance of Christensenellales CAG-138 was higher in AN as a new observation for this cohort, whereas the abundance of Ruminococcaceae and Lachnospiraceae was higher in HC. Among the 89 identified bacterial families, Christensenellaceae was the most significantly enriched in AN (Extended Data Fig. 2c). We observed higher *β*-diversity of AN microbiome at the genus level (Extended Data Fig. 2d), with *Bacteroides* being the dominant phylotype in both groups (Extended Data Fig. 2e). Among the top 30 genera, *Faecalibacterium, Agathobacter, Gemmiger*, unclassified Lachnospiraceae G, *Ruminococcus 2, Roseburia, Dysosmobacter – Oscillibacter, Coprococcus*, Oscillospirales 4 CAG-103 *and Eisenbergiella* were more abundant in HC, while unclassified Christensenellales CAG-138 was more abundant in AN (Extended Data Fig. 2e). Additionally, among the 225 identified bacterial genera, *Lactobacillus* was the most significantly enriched in AN (Extended Data Fig. 2f). Despite the difference in major genera between AN and HC, we found that richness of metagenomic species pangenomes (MSP, hereafter called species) was similar between the two groups (Extended Data Fig. 2g). In enterotype analyses^22^, we found a higher prevalence of the Ruminococcacea-enterotype (R-enterotype) in AN compared with HC, and a higher prevalence of the same enterotype in AN-BP than in AN-RS subtype (Extended Data Fig. 2h).

**Fig. 1:**
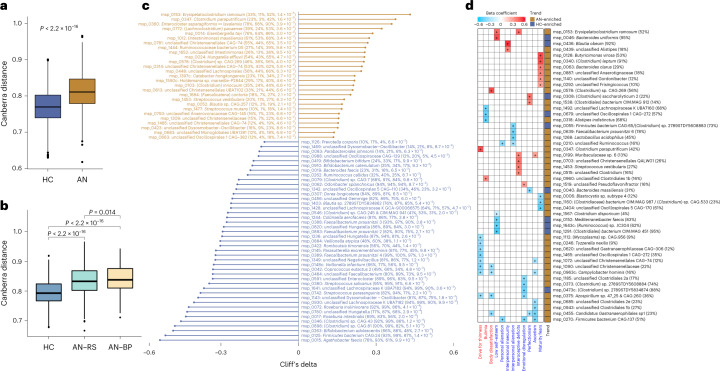
Alterations in gut bacterial species in AN cases compared to healthy controls, and associations with eating disorder scores. **a**,**b** Box plot (line, median; box, interquartile range (IQR); whiskers, 1.5× IQR) of β-diversity of AN (*n* = 77) and HC (*n* = 70) gut microbiota (**a**) and of two AN subtypes (AN-RS *n* = 56, AN-BP *n* = 21) and HC gut microbiota (**b**) at bacterial species level (Canberra distance). Statistical significance of differences between two groups was determined by Wilcoxon rank-sum test (two-sided). **c**, Significantly contrasted bacterial species between AN and HC. Differences in abundance were detected using the metadeconfoundR pipeline where covariates including age, BMI, smoking and multiple drug intake were corrected. Cliff’s delta values give estimates of effect size. For each contrasted MSP, prevalence in the whole cohort, HC, AN, and *P*_adj_ are given next to the MSP annotation. **d**, Heat map showing that gut bacterial species are linked to eating disorder scores in AN cases, using a linear regression model where age, BMI, smoking and multiple drug intake were defined as covariates and adjusted for. Variables in specific eating disorder scale are marked in blue, and general psychological scale is marked in red. Right panel to the heat map indicates the direction of each variable. For each MSP, prevalence in AN is given next to the MSP annotation. +, *P*_adj_ < 0.05 by Benjamini-Hochberg method (see for exact *P* values). Source data

**Extended Data Fig. 3:**
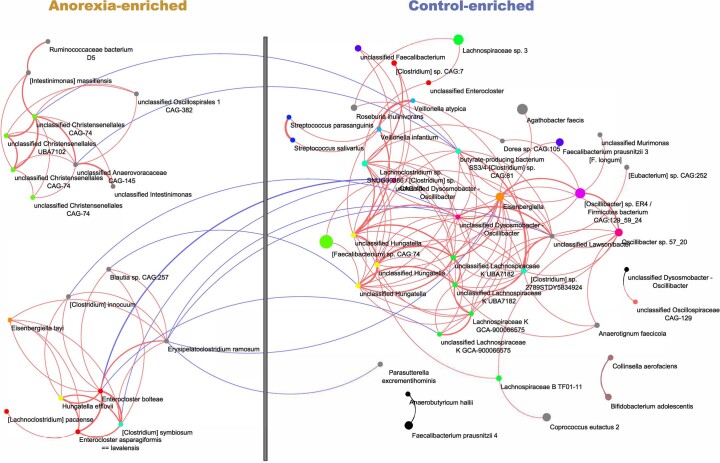
Co-occurrence network deducted from bacterial species enriched in AN cases (left) and healthy controls (right) after drug-deconfounding. Node size and node colour represent the mean abundance and the genus of a given MSP, respectively. Genera represented by only one MSP are coloured in grey. Red and blue lines indicate positive and negative correlations, respectively. Line thickness represents the absolute correlation coefficient. Only correlations with an absolute coefficient above 0.4 are shown; MSP without any correlation above the threshold are hidden.

At the species level (Supplementary Table 3), we observed that the AN gut microbiota is characterized by higher *β*-diversity (Fig. 1a). Within AN subgroups, the AN-BP subtype had a more heterogeneous bacterial community at the species level than the AN-RS subtype (Fig. 1b). In the comparison between in- and out-patient AN cases, we found that *β*-diversity of in-patients was higher than that of out-patients at the species level (Supplementary Fig. 1 and Data). Recent body weight change within 4 weeks was not associated with gut bacterial composition (Supplementary Table 4). The species that were significantly different in abundance distribution between AN and HC after deconfounding interferences of multiple medications (selective serotonin re-uptake inhibitors, antipsychotics and benzodiazepines, specified in Supplementary Table 2) are shown in Fig. 1c. Among the depleted species in AN were *Roseburia intestinalis* and *Roseburia inulinivorans*, species that have a high capacity for digesting plant polysaccharides and are considered to be part of the health-related gut microbiota^23^. In a co-abundance undirected network analysis (Extended Data Fig. 3), we identified a bacterial community consisting of *Eisenbergiella*, butyrate-producing bacterium SS3/4 - (*Clostridium*) sp. CAG:81, *Faecalibacterium prausnitzii* 3, (*Oscillibacter*) sp. ER4/*Firmicutes bacterium* CAG: 129_59_24, *Oscillibacter* sp. 57_20, (*Clostridium*) sp. 2789STDY5834924*, unclassified Lachnospiraceae* and unclassified *Dysosmobacter – Oscillibacter*, which was more abundant in HC. A community that was highly enriched in AN comprised *Erysipelatoclostridium ramosum*, *Enterocloster bolteae*, (*Clostridium*) *innocuum* and *Blautia* sp. CAG:257.

### Associations between absolute bacterial abundance and bioclinical variables in AN

**Fig. 2:**
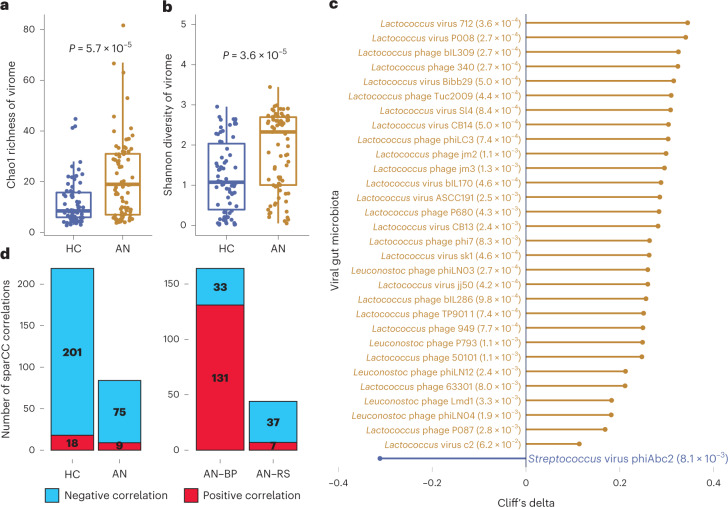
The viral gut microbiota differs between AN cases and controls. **a**,**b**, Box plot (line, median; box, IQR; whiskers, 1.5× IQR) of changes in Chao1 richness (**a**) and Shannon diversity (**b**) of the viral gut microbiota between AN (*n* = 77) and HC (*n* = 70) at viral species level. Significance was examined by two-sided Wilcoxon rank-sum test (**a**,**b**). **c**, Cliff’s delta values of contrasted gut viral species between AN and HC with *P*_adj_ < 0.05 by Benjamini-Hochberg correction (given next to the viral annotation). The differential species were identified by metadeconfoundR pipeline where impacts of cofactors including age, smoking and multiple drug intake were deconfounded. **d**, Difference in number of trans-kingdom ecologic correlations between the viral and bacterial gut microbiota in AN (*n* = 77) compared to HC (*n* = 70), and between two AN subtypes (AN-RS *n* = 56, AN-BP *n* = 21) using the SparCC algorithm. Source data

We investigated numerical covariations between the absolute abundance of bacteria at genus and species levels and bioclinical variables in the combined HC and AN cohort. We used a linear regression model adjusting for confounders including age, smoking and multiple drug intake (Methods and Supplementary Note 3, Fig. 2 and Data).

**Extended Data Fig. 4:**
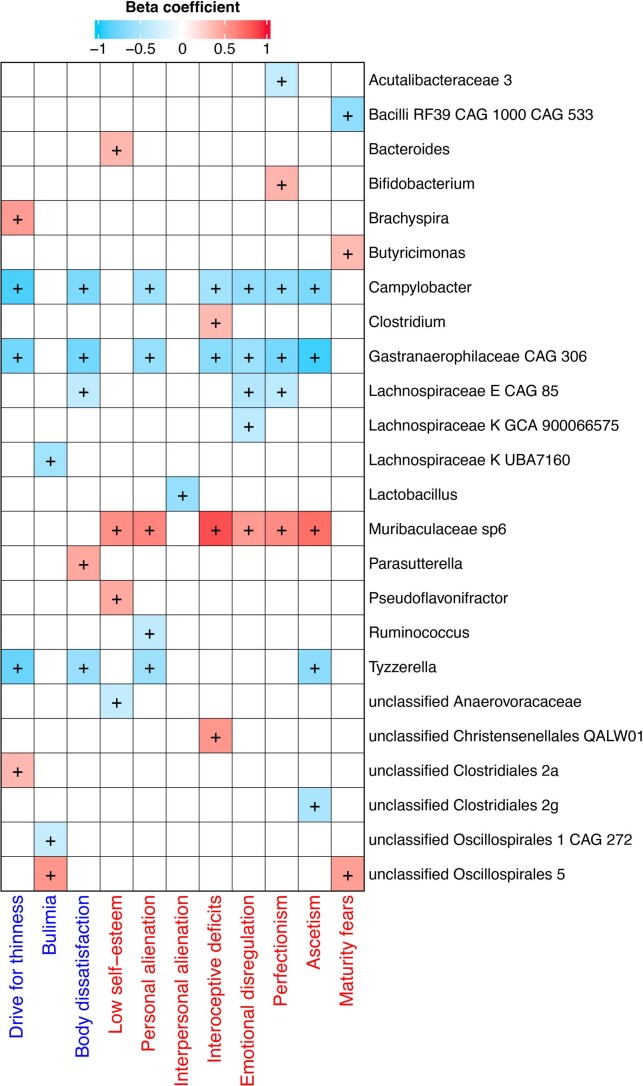
Heatmap showing that gut bacterial genera are linked to eating disorder scores in AN cases after drug de-confounding. Variables in specific eating disorder scale are marked in blue, and in general psychological scale are marked in red. +, adjusted p < 0.05 (see Source Data for exact p values). Source data

**Extended Data Fig. 5:**
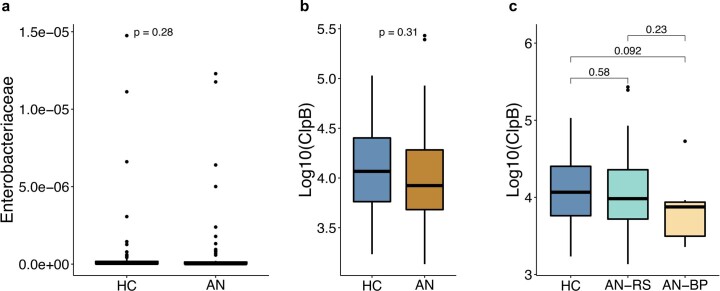
Fasting plasma concentration of Caseinolytic protease B (ClpB) in control subjects and AN subtypes. **a**, Relative abundance of Enterobacteriaceae family in HC (*n* = 70) and AN (*n* = 77) groups. **b**, Log-scale transformed fasting plasma ClpB concentration between HC (*n* = 70) and AN (*n* = 77) groups. **c**, Log-scale transformed fasting plasma ClpB concentration between restrictive AN (AN-RS *n* = 56) and binge-purge AN (AN-BP *n* = 21) subtypes. Significance was calculated by two-sided Wilcoxon rank-sum test between two groups (**a**-**c**). Box plots indicate median and interquartile range (IQR) and whiskers represent 1.5× IQR (**a**-**c**). Source data

Interestingly, some bacterial taxa were linked to eating disorder scores and psychological conditions after adjusting for multiple confounding factors including age, body mass index (BMI), smoking history and medications. At the species level, we found that *Clostridium* species were positively correlated with eating disorder scores (Fig. 1d), indicating a potential role of these species in the regulation of eating behaviour and neuropsychiatric symptoms^24^. Moreover, among the bacterial species that were inversely correlated with eating disorder scores, we found that the absolute abundances of *Lactococcus acidophilus*^25^ and *Faecalibacterium prausnitzii*^26^, both of which are associated with depressive symptoms, were related to a score for interpersonal alienation (Fig. 1d). Additionally, the absolute abundance of *Parasutterella* correlated positively with body dissatisfaction and absolute abundance of *Bifidobacterium* correlated with a marker of perfectionism. Despite a similar absolute abundance of *Brachyspira* in AN and HC, this genus was positively correlated with markers for ‘drive for thinness’ in AN (Extended Data Fig. 4). As shown in Extended Data Fig. 5, we found no difference in circulating levels of anorexigenic ClpB^14^ between AN and HC groups (Supplementary Note 4).

### Predicted bacterial growth rates are altered in AN

**Extended Data Fig. 6:**
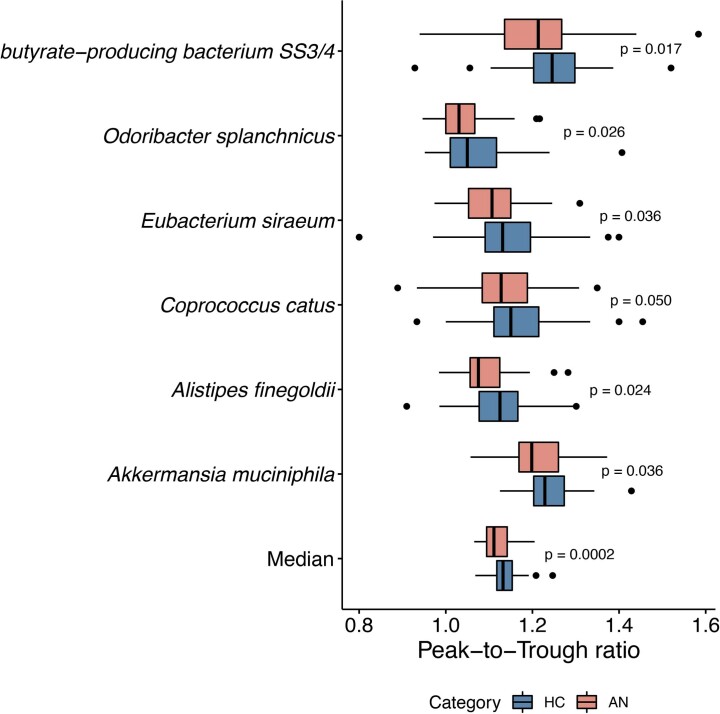
Altered dynamic growth rates of bacterial species between AN (*n* = 77) and control (*n* = 70) groups. Box plots indicate median and interquartile range (IQR), whiskers indicate 1.5× IQR. Significance was determined by two-sided Wilcoxon rank-sum test. Source data

We estimated growth dynamics of the bacterial gut microbiota from the metagenomic data by calculating the peak-to-trough ratio (PTR)^27^ for 50 bacterial species. Thirty-five of these were present in more than 20 samples. The median PTR values differed markedly between AN and HC (*P*_Wilcoxon_ = 2.0 × 10^−4^, Extended Data Fig. 6), which might be related to the severe reduction in food intake in AN patients. Six bacteria were predicted to have significantly lower growth rate in AN (*P*_Wilcoxon_ < 0.05, Extended Data Fig. 6). These were *Akkermansia muciniphila*, *Alistipes finegoldii*, *Coprococcus catus*, *Eubacterium siraeum*, *Odoribacter splanchnicus* and butyrate-producing bacterium SS3/4.

### *Lactococcus* phages and weakened trans-kingdom interactions in AN

We observed higher viral richness (Chao1, Fig. 2a) and diversity (Shannon, Fig. 2b) in AN faecal samples compared with HC. Recent body weight change within 4 weeks was not associated with gut viral composition (Supplementary Table 4). After deconfounding for covariates (age, smoking and drug intake), we identified 31 viral species that were enriched or decreased in AN (Fig. 2c). Of major interest, 25 of the 30 increased viral species in AN were *Lactococcus* phages with known *Lactococcus lactis* hosts—bacteria that have been extensively used in the production of fermented food products.

Analysis of viral–bacterial correlations within the AN and HC gut microbiota revealed a remarkable decrease in the number of viral–bacterial interactions in AN (219 for HC versus 84 for AN, *P*_Fisher’s exact test_ = 8.8 × 10^−15^, Fig. 2d). This was primarily driven by weakened interactions between viral species and short-chain fatty acid bacterial producers, such as *Roseburia inulinivorans*, *Faecalibacterium prausnitzii* and *Roseburia hominis* (Supplementary Fig. 3 and Data). We did not observe any interplay between *Lactococcus* phages and *Lactococcus* bacteria in the trans-kingdom analysis (Supplementary Figs. 3 and 4 and Data).

In analyses of AN subgroups, principal coordinate analysis (PCoA) on the Canberra distance showed no remarkable alterations in gut viral composition (*P*_PERMANOVA_ = 0.571, Supplementary Fig. 5 and Data). However, when comparing the number of viral–bacterial correlations in AN-RS and AN-BP gut microbiota, we found a reduction in the number and a much lower ratio of positive correlations in AN-RS (164 for AN-BP versus 44 for AN-RS, *P*_Fisher’s exact test_ < 2.2 × 10^−16^) (Fig. 2d and Supplementary Fig. 4). This suggests that the gut microbiota in AN-RS has weakened gut viral–bacterial interactions.

### Predicted gut microbiota functions correlate with eating behaviours and metabolism

**Fig. 3:**
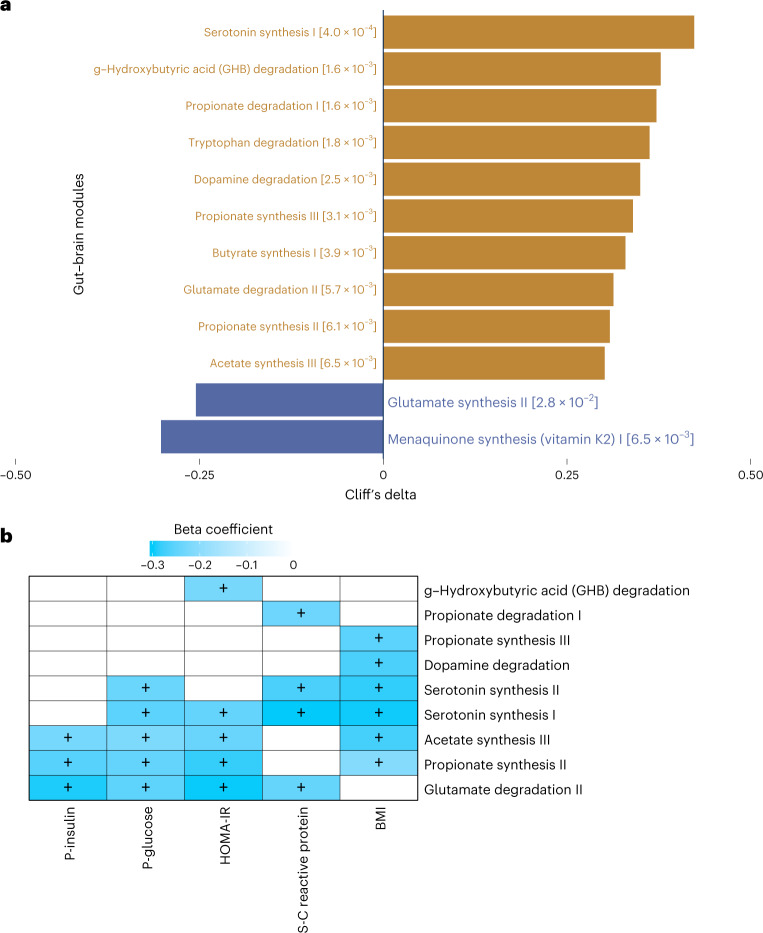
Predicted functional potentials of the bacterial gut microbiome in AN cases and healthy control participants. **a**, Cliff’s delta effect size of contrasted functional modules between AN (*n* = 77) and HC (*n* = 70) using the metadeconfoundR pipeline where interferences from covariates including age, BMI, smoking and multiple drug intake were corrected. Gold bars, functional modules more abundant in AN; blue bars, functional modules more abundant in HC. For each contrasted module, *P* value after Benjamini-Hochberg correction is given next to the module annotation. **b**, Heat map of the associations between clinical variables and functional potentials of gut bacteriome by linear regression model where impacts of covariates including age, smoking and multiple drug intake were deconfounded. + indicates *P* < 0.05 by Benjamini-Hochberg correction (see for exact *P* values). Source data

Using Gut Metabolic Modules (GMMs)^28^ and Gut Brain Modules (GBMs)^29^ to predict gut bacterial functional potentials, we identified 159 functional modules. Notably, the abundance of GBMs for serotonin biosynthesis and the degradation of dopamine, glutamate and tryptophan, which are metabolites with effects on mood and appetite, were enriched in AN (Fig. 3a). Conversely, the abundance of the glutamate synthesis II and vitamin K2 pathways were higher in HC (Fig. 3a)^30^. In addition, we found that serotonin synthesis and glutamate degradation pathways were inversely correlated with circulating concentrations of glucose and insulin, or insulin sensitivity (Fig. 3b). While we observed differences in GBMs, we did not identify differences in GMMs between AN and HC (Supplementary Table 5).

**Fig. 4:**
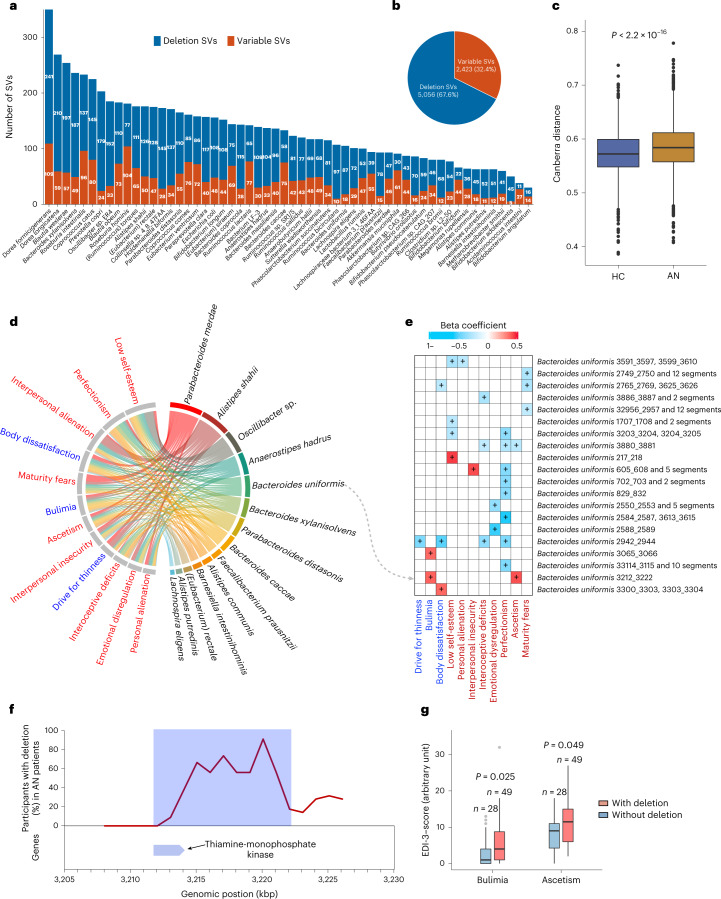
Structural variations in the bacterial and archaeal gut microbiota in AN cases and healthy controls. **a**, Number of SVs of each bacterial or archaeal species in 147 (77 cases and 70 controls) study participants. For each species, the number of deletion and variable SVs are given. **b**, Pie chart showing the total identified SVs numbers. **c**, Box plot (line, median; box, IQR; whiskers, 1.5× IQR) of *β*-diversity (Canberra distance) of SV-based genetic composition in AN (*n* = 77) and HC (*n* = 70) bacteriome. *P* value was determined by two-sided Wilcoxon rank-sum test. **d**, Chord diagram showing significant associations between eating disorder scores and bacterial SVs after adjusting for age, BMI, smoking and multiple drug intake. **e**, Heat map showing the associations between SVs of *Bacteroides uniformis* and EDI-3 scores using linear regression model where impacts of age, BMI, smoking and multiple drug intake were deconfounded. + indicates *P* < 0.05 corrected by Benjamini-Hochberg (see for exact *P* values). In **d** and **e**, variables of eating disorder scale are coloured in blue, general psychological scale are in red, and bacterial SVs are in black. **f**, The deletion rate of the 10-kbp deletion SV harbouring thiamine-monophosphate kinase in *B. uniformis* genome in the AN group. **g**, Box plot (line, median; box, IQR; whiskers, 1.5× IQR) showing the EDI-3 scores in anorexia individuals with (*n* = 49) and without (*n* = 28) the 10-kbp deletion. Significance was determined by Wilcoxon rank-sum test (two-sided). Source data

Bacterial genomes may have structural variants (SVs) that potentially interfere with functional genes impacting the interplay between microbes and their host^31^. Therefore, differences in the presence or abundance of SVs between otherwise identical bacterial strains may underlie critical phenotypic and functional differences^31,32^. Here we profiled SVs in all samples and identified 5,056 deletion SVs and 2,423 variable SVs across 56 bacterial species (Fig. 4a,b). For some species, we observed marked differences in copy number variation. We identified 87 deletion SVs and 18 variable SVs in *Bacteroides uniformis* across 134 individuals, 78 deletions and 15 variable SVs in *Faecalibacterium prausnitzii* in 124 individuals, and 110 deletion SVs and 55 variable SVs in *Parabacteroides distasonis* in 121 individuals. For the archaeal microbiota, we only identified SVs in *Methanobrevibacter smithii* in 13 individuals (Fig. 4a, and Supplementary Fig. 6 and Data). To explore potential differences in bacterial genetics, we further computed the Canberra distance of bacterial SV profiles between all 147 samples (Fig. 4c). AN and HC samples were significantly different in the *β*-diversity of SV composition (*P*_Wilcoxon_ < 2.2 × 10^−16^). Collectively, these results suggest the composition of bacterial SVs differs between the two groups.

**Extended Data Fig. 7:**
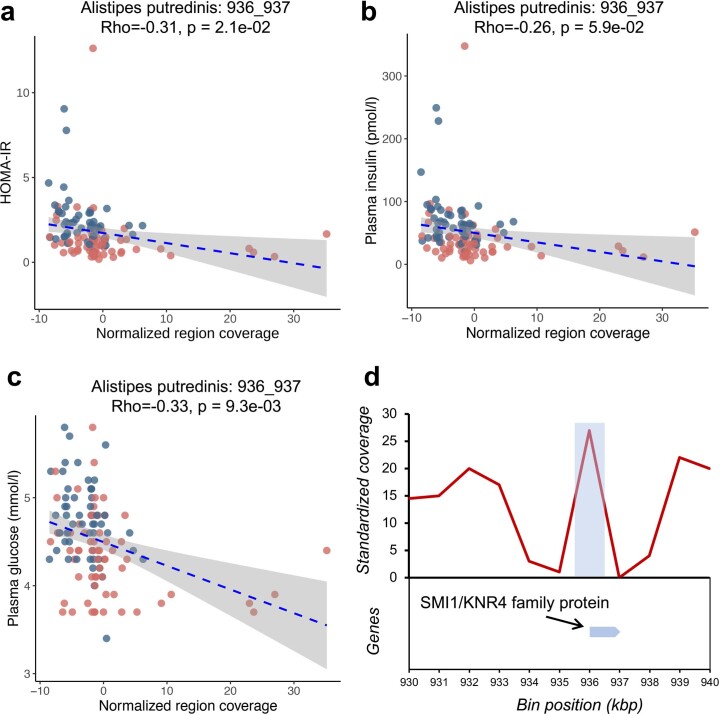
SVs in bacterial gut microbiota from AN cases and associations with anorexia-relevant traits. **a-c**, Scatterplot showing, HOMA-IR, fasting plasma insulin and glucose in individuals harboring a 1-kbp variation in the *A. putredinis* genome (*n* = 147). Significance determined by two-sided spearman correlation test corrected by false discovery rate using Benjamini and Hochberg method. Error band is linear regression line with 95% confidence band (**a**-**c**). Dots representing AN and HC individuals were colored in red and blue, respectively. **d**, Upper panel, standardized variability (y axis) along a genomic region of *A. putredinis* (x axis). Lower panel, locations (blue bar) of the gene of interest. Source data

Exploring relationships between gut bacterial SVs and markers of eating behaviour, we found that in AN cases, bacterial SVs were significantly associated with eating disorder scores after deconfounding for multiple covariates (Fig. 4d). As a noteworthy example, in the association analysis between SVs in the *B. uniformis* genome and host eating scores, we found that a 10-kbp deletion was directly associated with markers of bulimia and self-denial, indicating that this deletion SV may be involved in the regulation of eating disorder and psychological traits in AN (Fig. 4e). Indeed, gene analysis showed that the beginning of this specific genomic region in *B. uniformis* encodes a thiamine-monophosphate kinase (Fig. 4f and Supplementary Table 6), which is the distal enzyme involved in the thiamine (vitamin B1) biosynthesis pathway. Thiamine deficiency has been associated with mental health including memory loss, anxiety, depression, irritability, insomnia as well as appetite loss and gastrointestinal complaints^33^. About one third of AN cases may suffer from thiamine deficiency^34^. We found that AN cases lacking this bacterial genomic region had higher scores for bulimia (a key characteristic for the AN-BP subtype) and self-denial (Fig. 4g), a pattern also suggested by our correlation analyses (Fig. 4e). Another example linking bacterial genetics to metabolism-related bioclinical variables is given in (Extended Data Fig. 7) and Supplementary Note 5.

### Serum metabolites are associated with markers of appetite and food intake regulation

**Fig. 5:**
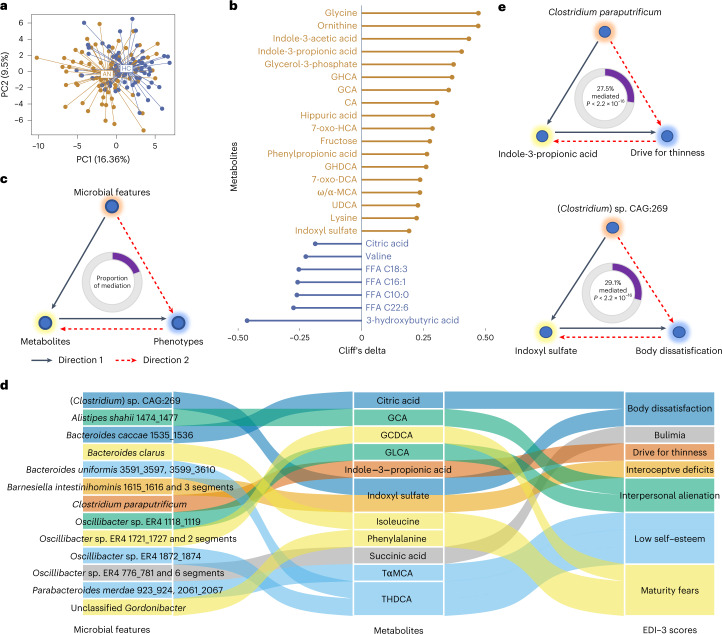
Serum metabolites differ between AN cases and controls and may be mediating the impact of gut microbial features on eating disorder traits. **a**, Principal component analysis (PCA) of the serum metabolome profile of AN cases and HC participants. **b**, Cliff’s delta values of contrasted metabolites between AN (*n* = 77) and HC (*n* = 70) after adjusting for age, BMI, smoking and multiple drug intake. Gold lollipops are metabolites enriched in AN, and blue lollipops show serum metabolites enriched in HC. **c**, Workflow for the bidirectional mediation analysis for gut microbial features, serum metabolites and host phenotypes. **d**, Sankey diagram showing the inferred causal relationship network of direction 1 where gut microbial features including bacterial species, gut brain/metabolic modules and bacterial genetics were treated as causal factors, metabolites are mediators, and EDI-3 scores are outcomes. **e**, Examples of inferred causal relationships between microbial features, metabolites and EDI-3 scores. Direction 1 means microbial features → eating disorder scores mediated by metabolites, illustrated with a black line; direction 2 means microbial features → metabolites mediated by EDI-3 scores, illustrated with a dashed red line. The proportions of mediation effects are shown at the centre of ring charts. FFA, free fatty acid. Source data

**Extended Data Fig. 8:**
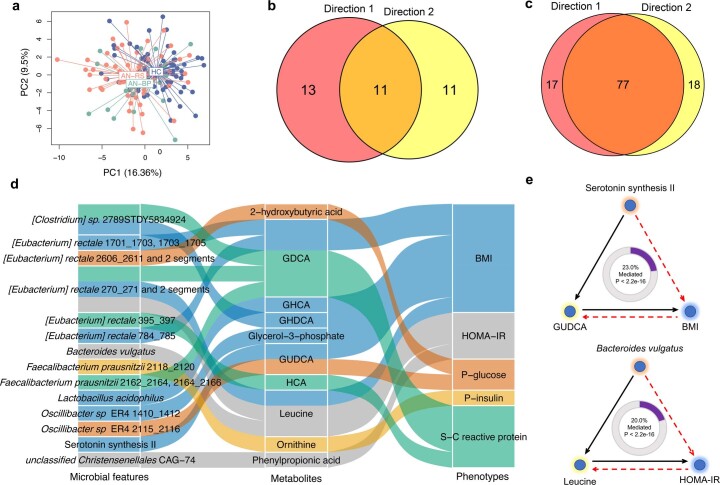
*In silico* analysis using bidirectional mediation inference. **a**, Principal component analysis (PCA) plot of the serum metabolome of AN subtypes, AN-BP (binge/purge anorexia), AN-RS (restrictive anorexia). **b**, Summary number of inferred mediation relationship for direction 1 (gut microbial features → eating disorder scores mediated by serum metabolites), direction 2 (gut microbial features → serum metabolites mediated by eating disorder scores). **c**, Summary number of inferred mediation relationship for direction 1 (gut microbial features → phenotypes mediated by serum metabolites), direction 2 (gut microbial features → serum metabolites mediated by phenotypes) for the whole cohort. **d**, Sankey diagram showing the inferred causal relationship network of direction 1 where gut microbial features including bacterial species, gut brain and metabolic modules, and bacterial genetics were treated as causal factors, serum metabolites are mediators, and metabolic traits are outcomes. **e**, Examples of inferred causal relationships between gut microbial features, metabolites, and host metabolic traits. Direction 1that means microbial features → metabolic traits mediated by serum metabolites is illustrated with a black line while direction 2 that means microbial features → serum metabolites mediated by metabolic traits is illustrated with a stipulated red line. The proportion of mediation effect are shown at the center of ring charts. GDCA, glycodeoxycholic acid; GHCA, glycohyocholic acid; GHDCA, glycohyodeoxycholic acid; GUDCA, glycoursodeoxycholic acid; HCA, hyocholic acid; HOMA-IR homeostatic model assessment of insulin resistance; P-, plasma; S-, serum. Source data

We performed untargeted metabolomics profiling of serum from AN cases and controls. This revealed a serum metabolome profile consisting of 28 polar metabolites and 35 microbiota-related metabolites, which was significantly different between AN and HC (Fig. 5a), while only slightly altered between the two AN subtypes (Extended Data Fig. 8a). We identified 25 serum metabolites with concentration differences between cases and controls after adjusting for confounders (adjusted *P* value (*P*_adj_) < 0.05) (Fig. 5b).

Serum concentrations of both primary (cholic acid (CA), glycocholic acid (GCA)) and secondary bile acids (glycohyocholic acid (GHCA), 7-oxo-hyocholic acid (7-oxo-HCA), glycohyodeoxycholic acid (GHDCA), 7-oxo-deoxycholic acid (7-oxo-DCA), ω/α-muricholic acid (ω/α-MCA), ursodeoxycholic acid (UDCA)) were higher in AN, indicating a potential role of the gut microbiota in AN-related changes in secondary bile acid synthesis and metabolism, and satiety regulation^35^. Moreover, serum concentrations of indole-3-acetic acid and indole-3-propionic acid (IPA), two tryptophan metabolites, were higher in AN compared with HC. Interestingly, IPA is associated with the secretion of glucagon-like peptide 1, which can stimulate satiety and slow gastric emptying^36,37^. Dysregulation of valine, saturated and long-chain unsaturated fatty acids were also observed in the AN group (Fig. 5b and Supplementary Note 6).

### Associations between serum metabolites, gut microbiota, and markers of appetite and mental health

To explore the role of serum metabolites in the interaction between gut microbiota and host phenotypes, we constructed bidirectional mediation models. For direction 1, we hypothesized that serum metabolites (including microbiota-related metabolites) as variables mediate the causal effect of gut microbial features (bacterial species, gut brain modules and bacterial genetics) on host phenotypes (Eating Disorder Inventory-3 (EDI-3) scores and metabolic traits). For direction 2, we treated phenotypes as mediators and alterations in metabolites as outcomes of changes in microbial features (Fig. 5c). This in silico bidirectional analysis enabled us to quantify the extent to which a hypothesized mediator (a metabolite) participates in the interaction between a cause (microbial features) and its effect (host phenotypic traits).

We first performed the causal inference of bacterial features on EDI-3 questionnaire scores in AN cases (Extended Data Fig. 8b). Inferred causal relationships in direction 1 consisted of 13 microbial features as initiators, 11 metabolites as mediators and 7 EDI-3 scores as outcomes (Fig. 5d). As a noteworthy example, we identified ‘drive for thinness’ as an outcome of the AN-enriched bacterial species *C. paraputrificum* (Fig. 1c), which was linked to changes in serum IPA levels, also enriched in the AN group. *C. paraputrificum* is a producer of multiple tryptophan catabolites, including IPA, indoleacrylic acid, indoleacetic acid and tryptamine, all of which are involved in regulating appetite and mental health^24^ (Fig. 5e).

In another example, indoxyl sulfate, which was enriched in AN patients, was identified as a mediator of AN-enriched (*Clostridium*) sp. CAG:269 in the score for body dissatisfaction (Fig. 5e). Indoxyl sulfate is a cardio- and uraemic toxin, and has been shown to induce anxiety- or depression-like behaviours in humans and animal models^38,39^. *Clostridium* is a genus of indole-producing bacteria that can encode tryptophanase^40^, the critical enzyme converting tryptophan to indole, pyruvate and ammonia^41^.

Next, we analysed causal inference between microbial features and host metabolic traits across the whole cohort (Extended Data Fig. 8c). The causal network in direction 1 consisted of 14 microbial features, 10 metabolites and 5 metabolic traits as causal treatments, mediators and outcomes, respectively (Extended Data Fig. 8d). Notably, we found that the serotonin synthesis module causally affected host BMI via the secondary bile acid glycoursodeoxycholic acid, which is upregulated by serotonin^42^ (Extended Data Fig. 8e). Finally, consistent with our previous findings, serum leucine mediated the impact of *B. vulgatus* on glucose homoeostasis^43^ (Extended Data Fig. 8e). We observed no unidirectional causal relationship between changes in eating behaviour and psychological status, gut microbial features and metabolites (Supplementary Table 7).

### Gut microbiota induces reduced weight gain and altered energy metabolism in mice

**Extended Data Fig. 9:**
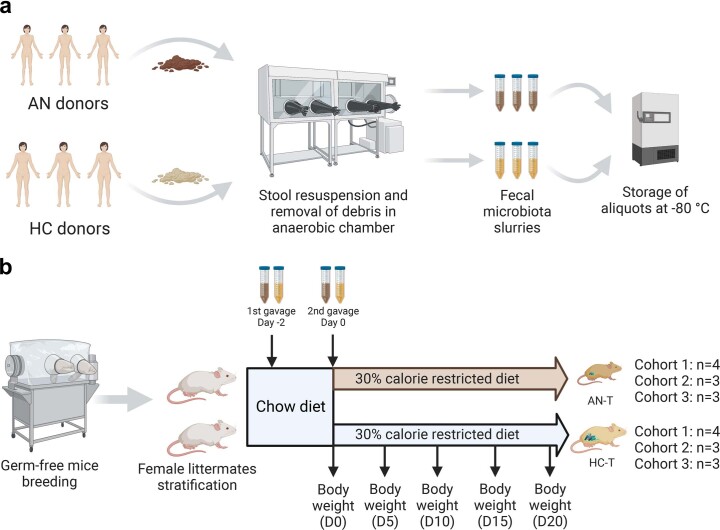
Workflow diagram of fecal microbiota transplantation from human donors to germ-free mice littermates. **a**, Workflow for the preparation of fecal microbiota slurry. Stool (250 mg) from both anorexia (AN) and control (HC) was cut on dry ice, transferred to anaerobic chamber, and resuspended with 5 ml of LYBHI media diluted in 20% glycerol. The resuspended fecal slurries were aliquoted in cryotubes and refrozen back quickly at -80 °C until further use. All stool samples of matched AN and HC donors were prepared on the same day and frozen aliquots were stored frozen for gavage to mice. **b**, Experimental scheme for the GF mice transplantation study. In each independent litter study, GF female littermates at age of six weeks old were taken out of breeding isolator and were randomly assigned to receive 200 µl of fecal slurries from three AN cases or three HC subjects. Both groups of mice were housed in autoclaved individually ventilated cages and were given autoclaved chow diet and water *ad libitum* for two days. After two days, mice were gavaged with a second dose of fecal material from the same matched AN and HC donors as before. Thereafter, mice in both groups were single housed and subjected to 30% calorie restricted chow diet for three weeks. Water was given *ad libitum* during this period. Both the anorexia-transplanted (AN-T) and the normal control-transplanted (HC-T) mice were weighed every five days after the start of energy-restricted diet. Created with Biorender.com.

**Extended Data Fig. 10:**
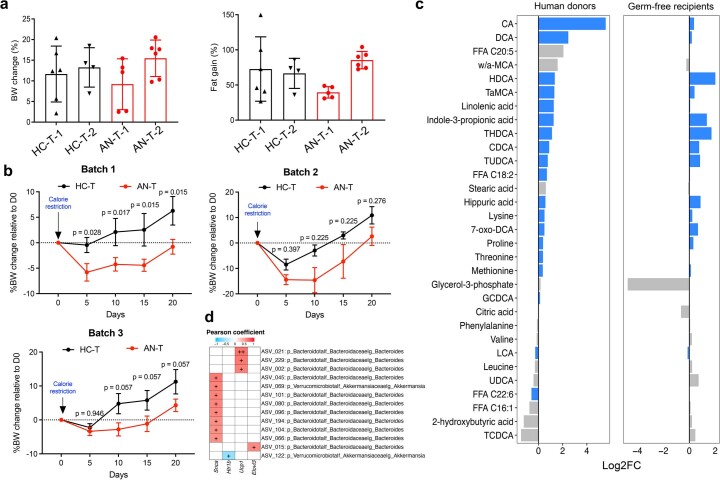
Effects of FMT from AN and controls to female GF mouse littermates. **a**, Body weight change and fat percentage of germ-free (GF) mouse littermates (*n* = 21) fed with *ad libitum* chow diet and transplanted with microbiota from anorexia patients. **b**, Body weight change compared to the body weight at day 0 after energy-restricted diet for three independent experiments (*n* = 8, 6, and 6 for independent batches, respectively). Data are expressed as mean ± s.e.m.(**a**,**b**). Significance was calculated by two-way analysis of variance (ANOVA), followed by Benjamini-Hochberg *post hoc* test. **c**, Serum metabolome profile in human donors and GF mouse recipients fed with 30% energy-restricted diet. Data were expressed as the log2-transformed fold changes (log2FC) between AN and control groups. Positive log2FC values indicate AN-enriched metabolites, while negative log2FC values indicate HC-enriched metabolites. Serum metabolites that were persistently changed between AN and HC groups in human donors and GF mouse recipients were marked with blue bars. CA, cholic acid; DCA, deoxycholic acid; FFA, free fatty acid; w/a-MCA, ω/α-muricholic acid; HDCA, hyodeoxycholic acid; TaMCA, taurine-α-muricholic acid; CDCA, chenodeoxycholic acid; LCA, lithocholic acid; UDCA, ursodeoxycholic acid; G, glycine-conjugated bile acids; T, taurine-conjugated bile acids. **d**, Heat map on the left panel showing the correlation between ASVs and quantified genes in hypothalamus or subcutaneous white adipose tissue. Taxonomic information of ASVs is given on the right panel. +, p < 0.05 corrected by Benjamini-Hochberg method (see Source Data for exact p values). Source data

To investigate potential causal relationships between an altered gut microbiota in AN and relevant phenotypes, we transplanted faecal microbiota from three randomly chosen AN-RS cases (to achieve uniformity as AN-BP has a more heterogeneous phenotype than the AN-RS subtype) and three age-matched HC participants to three independent litters of female germ-free (GF) mice (Extended Data Fig. 9a). To minimize variations in genetic background, we included littermates as control mice. In each litter study, 8, 6 and 6 littermates, respectively, were randomly assigned as recipients of AN or HC faecal microbiota. Following stool transplantation, recipient mice were singly housed and received a 30% calorie restricted diet for 3 weeks to mimic reduced food intake in human AN (Extended Data Fig. 9b). Ad libitum chow diet feeding did not generate any phenotypic alterations in GF mice^44^, an observation consistent with a previous report on kwashiorkor^45^ (Extended Data Fig. 10a).

**Fig. 6:**
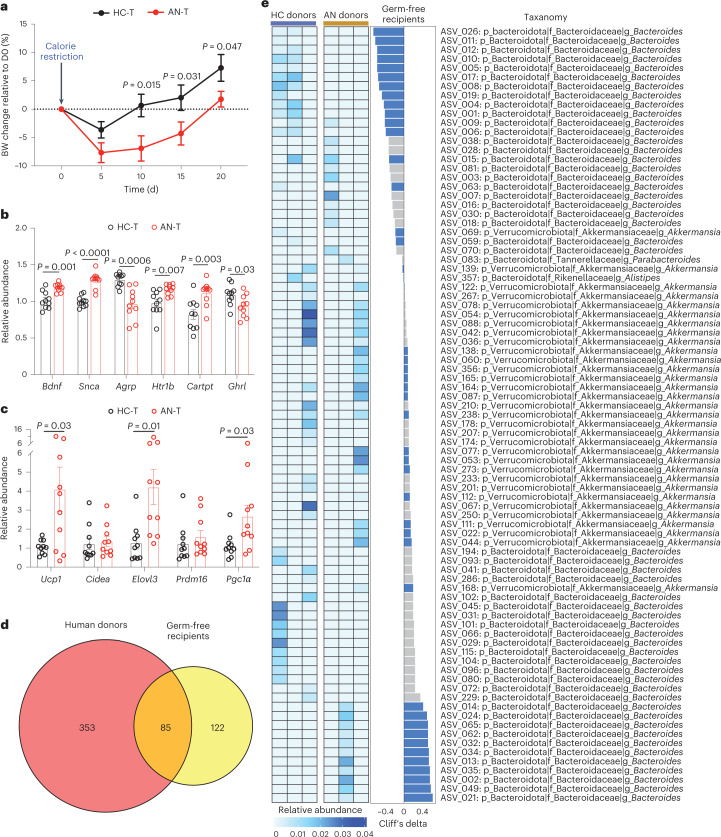
FMT using samples from AN donors induces AN-relevant phenotypes in GF mice. **a**, Body weight (BW) change compared to the body weight at day 0 after energy-restricted diet (AN-T *n* = 10, HC-T, *n* = 10 examined over 3 independent experiments). Significance was calculated by two-way analysis of variance (ANOVA), followed by Benjamini-Hochberg post hoc test. **b**,**c**, mRNA levels of the indicated mice genes in hypothalamus (**b**) and inguinal white adipose tissue (**c**) in the faecal microbiota mouse recipients (AN-T *n* = 10, HC-T, *n* = 10, examined over 3 independent experiments). Significance between the two groups was tested using unpaired two-tailed Student’s *t*-test. Data are presented as mean ± s.e.m. (**a**–**c**). **d**, Venn diagram of the identified and transferred ASVs between human donors and GF mouse recipients. **e**, Left: heat map of the 84 conserved ASVs in human donors. Middle: differences in the 84 conserved ASVs derived from the caecal content between AN-T and HC-T GF mouse recipients (AN-T *n* = 10, HC-T, *n* = 10, examined over 3 independent experiments). Transferred microbial alterations are marked in blue. Right: taxonomic information of ASVs. Source data

After 21 d, GF mice transplanted with stools from AN cases showed a larger initial decrease in body weight and a slower weight gain over time compared with mice that received HC FMT (Fig. 6a and Extended Data Fig. 10b; see Supplementary Note 7 for discussion).

We performed hypothalamic gene expression analysis following FMT. AN-transplanted and HC-transplanted recipients differed in expression of several hypothalamic genes involved in the control of eating behaviour and energy expenditure (Fig. 6b). This included increased expression of the appetite suppressors *Bdnf*^46^ and *Cartpt*^47^, and the receptor for serotonin, *Htr1b* (involved in the downstream regulation of serotonin), in AN FMT recipients. Expression of *Snca*, which encodes the neuronal protein alpha-synuclein associated with several neurodegenerative diseases, was higher in AN-transplanted mice^48^. In addition, we analysed messenger RNA (mRNA) levels of genes encoding proteins regulating adipose tissue thermogenesis. We found that abundances of *Ucp1*, *Elovl3* and *Pgc1α* mRNA were increased in inguinal fat of AN-transplanted mice, indicating enhanced adipose tissue thermogenesis in this group of mice (Fig. 6c).

16S ribosomal ribonucleic acid (rRNA) gene amplicon sequencing of human donor stools and GF mouse recipient caecal contents identified 85 overlapping amplicon sequence variants (ASVs; Fig. 6d), of which 45 (53%) altered ASVs in donors were transferred to recipients (Fig. 6e). Serum metabolome profiling detected 31 conserved metabolites between donors and recipients, and alterations of 19 of these (61%) were transferred from human to mouse (Extended Data Fig. 10c). We identified three ASVs: ASV_021, ASV_229 and ASV_002 that were annotated as *Bacteroides* at the genus level. The relative abundance of these ASVs and the expression of browning genes, including *Ucp1* (Extended Data Fig. 10d), were strongly positively correlated, indicating a potential role of these ASVs in body weight loss or lower weight gain through enhanced adipose browning. The inverse correlation between the relative abundance of ASV_122, annotated as genus *Akkermansia*, and the hypothalamic appetite-suppressing gene *Htr1b* is also notable and suggests a potential role of ASV_122 in appetite regulation (Extended Data Fig. 10d). Taken together, alterations in hypothalamic and adipose tissue gene expression and changes in body weight over time in mice suggest that a disrupted gut microbiota in human AN may contribute to some of the elements in the complex pathogenesis of AN.

## Discussion

Using a combination of sequencing and metabolomics to characterize the gut microbiome and metabolome in humans and mice, we show that the bacterial and viral components of the microbiome and the serum metabolome are altered in those with AN compared with healthy individuals. We also find that bacterial species SVs are different between AN and healthy controls, and in silico causal inference analyses imply that bacterial metabolites mediate some effects of an altered gut microbiota on AN behaviour. Finally, GF mice transplanted with AN stools on an energy-restricted diet initially lose more weight and have slower weight gain over time compared with mice transplanted with stools from healthy individuals. This was associated with higher expression of appetite suppressor genes in the hypothalamus and higher expression of thermogenesis-related genes in adipose tissue of AN-transplanted mice.

In both FMT experiments and in silico inference analyses, we observed changes in circulating levels of glycine-chenodeoxycholic acid, indole-3-propionic acid, taurine-α-muricholic acid and taurine-hyodeoxycholic acid. We propose that these metabolites may act as potential mediators of some of the AN phenotypes. For example, indole-3-propionic acid is a tryptophan metabolite, which is implicated in serotonin activity^49^, and hyodeoxycholic acid is a 6α-hydroxylated bile acid, also called muricholic acid, which reduces body weight gain^50^. This bile acid is involved in satiety regulation^51^. Serotonin activity as well as appetite regulation could be implicated in development and/or maintenance of the AN syndrome. Future studies will need to explore the individual and combined effects of these metabolites on energy metabolism. Still, many more gut bacterial species and derived metabolites may mediate the observed AN traits in humans and mice, as reported in an activity-based anorexia model^20,52^.

Analyses of deletion and variable SVs in gut bacterial species indicated that bacterial genetics may influence AN-relevant behavioural traits and pathophysiology. Of special interest is a 10-kbp deletion in the *B. uniformis* genome that was associated with estimates of bulimia and self-denial. We predicted that this deletion results in the loss of a thiamine-monophosphate kinase encoding gene, which may result in a relative deficiency of microbiota-produced thiamine. This is of interest in the context of AN pathology since various mental and intestinal health issues are known to be linked to thiamine deficiency^33^.

Our studies of the viral gut microbiota in AN showed a partial uncoupling of the ecological interactions between viral species and short-chain producing bacterial species with impact on brain biology^53^. In addition, AN samples were enriched in *Lactococcus* phages with known bacterial *L. lactis* hosts. The AN-associated enrichment in *Lactococcus* phages may result in disruption in food fermentation and suggest the possible use of fermented food items in future treatment of AN. The reason for this enrichment of *Lactococcus* phages is unsettled but our finding may justify testing a multistrain probiotic containing *L. lactis* in AN adolescents^54^.

Our study has limitations: (1) the use of a cross-sectional Danish AN cohort limits generalizability of findings to other ethnicities; (2) the same limitation holds for the use of AN patients in treatment in a specialized centre, as these patients may not be representative of milder forms of AN; and (3) although effects of multiple covariates were deconfounded, we had no information on diet and physical activity—behaviours that impact the gut microbiota.

In conclusion, the present multi-omics study uncovers profound and complex disruptions of the gut microbiota in individuals with AN, with functional implications and altered serum metabolites. These compounds may act via the blood circulation or via gut-microbiota-brain neuronal signalling pathways affecting brain regulation of appetite, emotions and behaviour. FMT from human AN donors to GF mice under energy-restricted feeding resulted in lower body weight gain and a number of changes in expression of hypothalamic and adipose tissue genes involved in controlling behaviour and energy homoeostasis. The combination of multi-omics and in vivo experiments complement our causal inference analyses to allow the identification of specific bacterial metabolites that potentially mediate human host AN traits. Our findings lend support to the hypothesis that a severely disrupted intestinal microbiota contributes to some of the stages in the pathogenesis of AN.

## Methods

### Study participants

The AN patients were all diagnosed by an experienced consultant psychiatrist and they met the DSM-5 criteria for the restricting or binge/purging subtypes of AN^1^. Since 90–95% of individuals with diagnosed AN are females, we decided to include only women in the present study and since ethnicity may influence gut microbiota, we only included Danish Caucasian women with AN cases, recruited from three specialized centres in Denmark from 1 September 12014 to 31 July 2016. The centres were: Center for Eating Disorders (Odense University Hospital), Child and Adolescent Psychiatric Unit (Aarhus University Hospital) and Unit for Psychiatric Research (Aalborg University Hospital). Exclusion criteria comprised antibiotic or antifungal treatment within the previous 3 months, any acute or chronic somatic diseases or infections. All the included patients were treated in specialized centres, and they were interviewed by an experienced and specialized psychologist or psychiatrist at the start of their treatment. The validated Eating Disorder Inventory (EDI, details given in Supplementary Note 2) was used for the interview and as a questionnaire filled out by trained health professional specialists^21^. The exclusion criteria for the age-matched healthy control women were BMI below 18.5 or above 25 kg m^−2^, regular medication of any kind apart from birth control pills, and antibiotics within the last 3 months. The control participants were recruited via public advertisement and via direct contact to health staff, medical students and their relatives. BMI and other clinical characteristics of the healthy controls are listed in Supplementary Table 2.

The study protocol was registered at ClinicalTrials.gov (NCT02217384) and the study was conducted in accordance with the Helsinki declaration and approved by the Regional Scientific Ethical Committee for Southern Denmark (file no 42053 S-20140040). All participants involved in this study provided written informed consent.

In-patients. All the below-described measurements were conducted during routine treatment in the specialized unit for somatic and psychological stabilization of patients with severe AN. Safe and effective weight restoration of 2.0–3.0% per week is the goal of the treatment in the inpatient unit. Care was given by a multidisciplinary team and is in accordance with international guidelines. Individually customized meals were given under the supervision of trained nurses or dietitians at scheduled times. If the meals could not be consumed within the predefined timeframes (15 min for a snack and 30 min for a main meal), supplemental nutrition drinks were added either orally or via a duodenal tube. To account for individual preferences, a choice of three different meals was offered. The macronutrient content was consistent and within recommended energy percent ranges: 40–50% carbohydrate (maximum of 10% sugar), 30–40% fat and 20–25% protein. The daily energy intake was individualized according to the weight course. If a patient failed to reach 2% of weekly weight gain, the energy content of the daily menu was increased. All meals were followed by a supervised rest varying from 30 to 60 min in a seated position. Between the rests, light physical activity such as a walk was allowed; however, no forms of exercise training were allowed. For patients with an urge for excessive exercise or otherwise a lack of compliance with behaviour rules, behaviour supervision was extended and if needed, to 24 h a day.

Out-patients. AN patients paid regular visits to outpatient units where care was given by a multidisciplinary team involving medical doctors, nurses, psychologists and health behaviour educators.

They were given an individual diet plan, which had the same macronutrient composition as mentioned above for in-patients. As with the in-patients, all out-patients were also enrolled in cognitive behavioural therapeutic treatment courses.

Height was measured on a wall-mounted stadiometer and weight was measured in the morning before breakfast on a calibrated platform scale. BMI was calculated as weight divided by the square of height (=kg/m^2^).

### Biochemical analyses

Blood samples were taken in the morning after an overnight fast. The blood samples were collected on ice and processed to obtain serum and plasma, and subsequently stored at −80 °C. Serum concentrations of sodium, potassium, albumin and creatinine were measured by enzymatic assays on a Roche/Hitachi cobas c system. Serum concentrations of total cholesterol, high-density lipoprotein cholesterol and low-density lipoprotein cholesterol were determined using the phosphotungstic acid magnesium chloride precipitation method. Serum immunoreactive insulin levels were measured using an enzyme-linked immunosorbent assay, while serum concentration of alanine aminotransferase was analysed with an enzymatic coulometric method including pyridoxal phosphate activation.

Analysis of plasma ClpB was performed as previously described^55^.

### Faecal sample collection and DNA extraction

Stools were collected according to International Human Microbiome Standards (IHMS) guidelines (SOP 03 V1) in kits by AN cases and HC at home and immediately frozen at −20 °C until they were transported on dry ice and frozen 4–24 h later at −80 °C in plastic tubes at the biobanks. DNA extraction from aliquots of faecal samples was performed following IHMS SOP P7 V2^56^.

### Bacterial cell counting

For bacterial cell counting (Supplementary Fig. 7), 0.08–0.12 g of frozen (−80 °C) faecal samples were diluted 15 times in pH 7.2 Dulbecco’s phosphate-buffered saline (DPBS) (Sigma-Aldrich), mechanically homogenized using tissue lyser (40 min, 12.5 agitations per second; QIAGEN) and fixed with 2% paraformaldehyde (10 min, room temperature; Biotum). Then the samples were diluted 120 times in filtered staining buffer (1 mM EDTA, 0.01% Tween20, pH 7.2 DPBS, 1% BSA (Sigma-Aldrich)). To minimize clumps, the samples were filtered through a cell strainer (pore size 5 μm; pluriSelect), pre-wet in the staining buffer. Next, the bacterial cell suspension was stained with SYBR Green I (1:200,000; Thermo Fisher) in DMSO (Sigma-Aldrich) and incubated in the dark for 30 min. For accurate determination of bacterial cell counts, a known concentration of 123count eBeads (Invitrogen) was added to the samples before the analysis. Measurements were performed using a BD Fortessa LSRII flow cytometer (BD Biosciences) and data were acquired using BD FACSDiVaTM software. A threshold value of 200 was applied on the FITC (530/30 nm) channel. Fluorescence intensity at green (530/30 nm, FITC), blue (450/50 nm, Pacific Blue), yellow (575/26 nm, PE) and red (695/40 nm, PerCP-Cy5-5) fluorescence channels as well as forward- and side-scattered (FSC and SSC) light intensities were collected. Measurements were performed at a pre-set flow rate of 0.5 μl s^−1^. Data were processed in R using flowcore package (v1.11.20)^57^ in R (v4.1.2). Fixed gating strategy separated the microbial fluorescent events from the faecal sample background.

### Shotgun sequencing

DNA was quantitated using Qubit fluorometric quantitation (Thermo Fisher) and qualified using DNA size profiling on a fragment analyser (Agilent). High molecular weight DNA (>10 kbp; 3 µg) was used to build the library. Shearing of DNA into fragments of approximately 150 bp was performed using an ultrasonicator (Covaris) and DNA fragment library construction was performed using the Ion Plus Fragment Library and Ion Xpress Barcode Adapters kits (Thermo Fisher). Purified and amplified DNA fragment libraries were sequenced using the Ion Proton Sequencer (Thermo Fisher), with a minimum of 20 million high-quality reads of 150 bp (on average) generated per library.

### Construction of a gene count table

To construct the gene count table, METEOR software was used^58^: first, reads were filtered for low quality by AlienTrimmer^59^. After removal of low-quality reads and human DNA reads, 75.7% ± 2.7% high-quality metagenomics sequencing reads of faecal DNA were mapped onto the Integrated Gut Catalog 2 (IGC2)^60^, comprising 10.4 million genes, using Bowtie2 (ref. ^61^). Reads mapped to a unique gene in the catalogue were attributed to their corresponding genes. Then, reads that mapped with the same alignment score to multiple genes in the catalogue were attributed according to the ratio of their unique mapping counts to the captured genes. The resulting count table was further processed using the R package MetaOMineR v1.31^62^. It was downsized at 14 million mapped reads to take into account differences in sequencing depth and mapping rate across samples. Then the downsized matrix was normalized for gene length and transformed into a frequency matrix by fragments per kilobase of transcript per million fragments mapped normalization. Gene count was computed as the number of genes present (abundance strictly positive) in the frequency matrix.

### Profiling and annotation of MSPs and gut enterotypes

IGC2 was previously organized into 1,990 MSPs with MSPminer^63^ using a publicly available updated MSP dataset^64^. Relative abundance of MSP was computed as the mean abundance of its 100 ‘marker’ genes (that is, the genes that correlate the most altogether). If less than 10% of ‘marker’ genes were seen in a sample, the abundance of the MSP was set to 0. This approach was used in the MetaHIT^62^ and Metacardis^65^ consortia. For the 4 MSPs with less than 100 core genes, all available core genes were used.

Abundances at higher taxonomical ranks were computed as the sum of the MSP that belong to a given taxa. MSP count was assessed as the number of MSPs present in a sample (that is, whose abundance is strictly positive). Enterotypes profiling was performed as previously demonstrated^66^.

### Estimating functional modules of gut bacteriome

Genes from the IGC2 catalogue were mapped with diamond^67^ onto KEGG orthologues (KO) from the KEGG database^68^ (v8.9). Each gene was assigned to the best-ranked KO among hits with *e*-value < 10 × 10^−5^ and bit score >60. Then we assessed presence and abundance of GMMs^28^ and GBMs^29^ in a metagenomic sample by the pipeline implemented in the R package omixerRpm (v0.3.2) as previously described^28,29^.

### Estimation of dynamic growth rate of bacteria from metagenomic samples

We used the computational pipeline to infer gut bacterial growth dynamics from metagenomic samples as previously described^27^. Sequencing reads were mapped to a database that contains complete genomic references of 2,991 strains belonging to 1,509 microbial species. For each bacterial species, a reference strain with prevalence of 100% across the samples was selected. A coverage map was then assembled on the basis of aligned reads to the reference genome. Genomic segments were binned into 10-kbp regions and coverage of the resulting bins was calculated and smoothed. The location of the origin and terminus of replication was predicted by fits of the same strain across multiple samples. Lastly, PTRs were calculated for each bacterial species in every sample as the smoothed sequencing coverage of the representative strain at the predicted peak location, divided by that at the predicted trough location.

### Studies of bacterial structural variations

Before the SVs classification, the pipeline with iterative coverage-based read assignment algorithm was performed to reassign the ambiguous reads to the most likely reference with high accuracy^31,32^. The reference genomes provided in the proGenomes database (http://progenomes1.embl.de/) were concatenated and then divided into genomic 1-kbp bins and applied for the detection of highly variable genomic segments. The SGV-Finder pipeline^31^ was used to detect the SVs that are either (1) with deletion percentage of the genomic segment across the population of <25% (variable SVs, vSVs), (2) with deletion percentage between 25% and 75% (deletion SVs, dSVs; the absence or presence of this particular genomic segment was kept) or (3) with deletion percentage of >75% (this genomic segment was excluded from the analysis). All bacterial species with SV calling were present in at least 10% of the total samples and were used for subsequent analysis.

### Mediation analysis

The R package ‘mediation’^69^ (v4.5.0) was used to infer causal relationships between gut microbial features, polar and microbiota-related metabolites, and metabolic traits and eating disorder scores. To reduce the testing numbers, we only kept the candidate groups consisting of variables that were strongly associated with each other; that is, for a candidate microbial feature-metabolite-phenotypic variable causal group, the association between gut microbial feature and serum metabolite was significant (*P*_adj_ < 0.1); the association between metabolite and phenotypic variable was significant (*P*_adj_ < 0.1); and the association between microbial feature and phenotypic variable was significant (*P*_adj_ < 0.1). After performing mediation analysis, only candidate groups with significance in direction 1 were kept for Sankey diagram visualization.

### Profiling and analysis of viral gut microbiota

We profiled the viral gut microbiota using MiCoP^70^, as this method is optimized to call viruses directly from bulk metagenomics sequencing reads and compute relative abundance within the virome dataset. As a reference dataset, MiCoP draws upon the NCBI’s RefSeq Viral database^71^. We identified a total of 209 viral species with prevalence of > and =10% and relative abundance of > and =0.01% for 147 (77 AN versus 70 HC) individuals included in the dataset. Richness, alpha and beta diversity were calculated with the R package ‘fossil’^72^ and ‘vegan’^73^. Two-tailed Wilcoxon’s rank-sum test was used to determine statistically significant differences in richness and alpha diversity indices between groups. Permutational multivariate analysis of variance (PERMANOVA) at *n* = 999 was performed for Canberra distance. The viral–bacterial interactions in both AN-RS and AN-BP microbiome data were computed using the Sparse Correlations for Compositional (SparCC)^74^ algorithm. Before the SparCC analysis, the AN bacterial and viral microbiota datasets were subset to AN-RS and AN-BP datasets, which were then separately submitted for SparCC analysis.

### Analysis of serum polar metabolites by gas chromatography–time-of-flight mass spectrometry

The metabolites listed as gut microbiota-related metabolites were based on literature mining^75,76^. Serum samples were randomized and sample preparation was done as described previously^43,77^. Briefly, 400 μl of methanol (MeOH) containing internal standards (heptadecanoic acid, deuterium-labelled dl-valine, deuterium-labelled succinic acid and deuterium-labelled glutamic acid, 1 µg ml^−1^) was added to 30 µl of the serum samples, which were then vortex mixed and incubated on ice for 30 min. Samples were then centrifuged (9,400 × *g*, 3 min) and 350 μl of the supernatant was collected after centrifugation. The solvent was evaporated to dryness, 25 μl of MOX reagent was added and the sample was incubated for 60 min at 45 °C. *N*-Methyl-*N*-(trimethylsilyl)trifluoroacetamide (25 μl) was added and after 60 min incubation at 45 °C, 25 μl of the retention index standard mixture (n-alkanes, 10 µg ml^−1^) was added.

The analyses were done using an Agilent 7890B gas chromatograph coupled to an Agilent 7200 quadrupole time-of-flight mass spectrometer. The following parameters were used: injection volume was 1 µl with 100:1 split on PTV at 70 °C, heating to 300 °C at 120 °C min^−1^; column: Zebron ZB-SemiVolatiles with length of 20 m, inner diameter of 0.18 mm, film thickness of 0.18 µm, with initial helium flow of 1.2 ml min^−1^, increasing to 2.4 ml min^−1^ after 16 min. Oven temperature programme: 50 °C (5 min), then to 270 °C at 20 °C min^−1^ and then to 300 at 40 °C min^−1^ (5 min). EI source: 250 °C, 70 eV electron energy, 35 µA emission, solvent delay 3 min. Mass range 55 to 650 amu, acquisition rate 5 spectra per second, acquisition time 200 ms per spectrum. Quad at 150 °C, 1.5 ml min^−1^ N_2_ collision flow, aux-2 temperature 280 °C.

Calibration curves were constructed using alanine, citric acid, fumaric acid, glutamic acid, glycine, lactic acid, malic acid, 2-hydroxybutyric acid, 3-hydroxybutyric acid, linoleic acid, oleic acid, palmitic acid, stearic acid, cholesterol, fructose, glutamine, indole-3-propionic acid, isoleucine, leucine, proline, succinic acid, valine, asparagine, aspartic acid, arachidonic acid, glycerol-3-phosphate, lysine, methionine, ornithine, phenylalanine, serine and threonine purchased from Sigma-Aldrich at a concentration range of 0.1–80 μg ml^−1^. An aliquot of each sample was collected, pooled and used as quality-control samples, together with National Institute of Standards and Technology (NIST) CRM1950 serum sample, an in-house pooled serum sample. The relative standard deviation of the concentrations was on average 16% for the pooled quality-control samples and 10% for the NIST samples.

### Analysis of serum bile acids and serum semipolar metabolites

The sample preparation procedure was performed as described previously^78^. The plate was preconditioned with 450 µl acetonitrile before the addition of 100 µl of sample and 10 µl of polyfluoroalkyl substances (PFAS) and bile acids (BAs) internal standard mixture (200 ng ml^−1^ and 1,000 ng ml^−1^, respectively). Thereafter, 450 µl of acetonitrile containing 1% formic acid were added to each well and the samples extracted using a 10″ vacuum manifold. The eluate was evaporated to dryness under nitrogen gas flow and reconstituted to 80 µl of MeOH/2 mM aqueous ammonium ethanoate.

Chromatographic separation was carried out using an Acquity UPLC BEH C18 column (100 mm × 2.1 mm inner diameter, 1.7 µm particle size), fitted with a C18 precolumn (Waters). Mobile phase A consisted of H_2_O:MeOH (v/v 70:30) and mobile phase B of MeOH with both phases containing 2 mM ammonium acetate as an ionization agent. The flow rate was set at 0.4 ml min^−1^ with the elution gradient as follows: 0–1.5 min, mobile phase B was increased from 5% to 30%; 1.5–4.5 min, mobile phase B was increased to 70%; 4.5–7.5 min, mobile phase B was increased to 100% and held for 5.5 min. A post-time of 5 min was used to regain the initial conditions for the next analysis. The total run time per sample was 18 min. The dual electrospray ionization source settings were as follows: capillary voltage was 4.5 kV, nozzle voltage 1,500 V, N_2_ pressure in the nebulizer was 21 psi and the N_2_ flow rate and temperature as sheath gas were 11 l min^−1^ and 379 °C, respectively. To obtain accurate mass spectra in the mass spectrometry (MS) scan, the *m*/*z* range was set to 100–1,700 in negative ion mode. MassHunter B.06.01 software (Agilent) was used for all data acquisition.

Identification of compounds was done with an in-house spectral library using MS (and retention time), tandem mass spectrometry information. Quantitation was based on a matrix-matched calibration curve spiked with native compounds. The calibration curve consisted of concentrations ranging from 0–1,600 ng ml^−1^ for BAs. The relative standard deviation for the BAs was on average 17.8% for the quality-control samples and 19.4% for the NIST samples.

### Animal experiments

Animal protocols were approved by the Science Ethics Committees of the Capital Region of Copenhagen, Denmark. Female germ-free Swiss Webster mice were bred and maintained in flexible film gnotobiotic isolators until the start of experiments at the Department of Experimental Medicine, University of Copenhagen. The mice were fed autoclaved chow diet (7% simple sugars, 3% fat, 50% polysaccharide, 15% protein (w/w), energy 3.5 kcal g^−1^) and water ad libitum under a 12 h light/12 h dark cycle (lights on at 7:30 a.m.) and constant temperature (21–22 °C) and humidity (55 ± 5%).

Faecal samples from randomly selected subsets of three patients with AN (females aged 20, 22 and 20 yr with BMI of 10.3, 11.5 and 11.7 kg m^−2^, respectively) and three HC (females aged 20, 22 and 21 yr with BMI of 22.6, 21.1 and 21.2 kg m^−2^, respectively) who were representatives of cases and controls were used to colonize 6-week-old female GF littermates. Briefly, 250 mg of faecal samples were suspended with 5 ml of LYBHI media (supplemented with 0.05% Cysteine and 0.2% Hemin as reducing agents) diluted in 20% glycerol (20 ml g^−1^ of faeces) in an anaerobic cryovial; these inoculum samples were then vortexed for 5 min, followed by 5 min standing to precipitate particles. The faecal slurries were then transferred to 1 ml cryovials and immediately frozen at −80 °C. At day one, both groups of mice were housed in autoclaved individually ventilated cages where they received the first dose (200 µl) of faecal slurries. The mice were then given autoclaved chow diet and water ad libitum for 2 d and their food intake was recorded. At day 3, mice were gavaged with a second dose of faecal material from the same matched AN and HC donors as before. Thereafter, mice in both groups were singly housed and subjected to 30% energy-restricted autoclaved chow diet (amount of given food was set at 70% of ad libitum food intake for each mouse) for 3 weeks where water was given ad libitum. Both the anorexia-transplanted (AN-T) and the normal control-transplanted (HC-T) mice were weighed every 5 d after the start of energy-restricted dieting.

At the end of the study, mice were anaesthetized with isoflurane and blood from the vena cava was collected in tubes containing EDTA. Blood samples were centrifuged for 6 min at 4,032 *g* at 4 °C. Plasma was isolated and stored at −80 °C for subsequent biochemical testing. Inguinal subcutaneous white adipose tissue, the caecum and the hypothalamus of each mouse were precisely dissected and collected for quantitative PCR analysis. No animal or data points were excluded from the present study.

Total RNA was extracted from tissues using Trizol reagent (Invitrogen) according to the manufacturer’s instructions, followed by concentration measurement. One µg of RNA was transcribed to complementary DNA using the Reverse Transcription System (Promega). Real-time PCR was performed using the LC480 detection system (Roche Diagnostics) and SYBR Green I Supermix (Takara). All qPCRs were run on the thermal cycles at 95 °C for 10 min, followed by 45 cycles of 0.01 s at 95 °C and of 20 s at 60 °C. Data were normalized to the housekeeping Rpl36 gene for adipose tissue and Rplp0 gene^79^ for hypothalamus and analysed according to the delta-delta CT method. Sequences of oligonucleotides used in this study are provided in Supplementary Table 8.

### DNA extraction, 16S rRNA sequencing and data analyses in mice experiments

Microbial DNAs were isolated and purified from stool samples (~250 mg) of human donors and mouse recipients by using NucleoSpin soil mini kit (MACHEREY‑NAGEL). The DNA was then amplified using Phusion High-Fidelity PCR master mix (New England Biolabs) by PCR targeting the V3-V4 region of the 16S rRNA gene (primer sequences provided in Supplementary Table 8). The following PCR programme was used: 98 °C for 30 s, 25× (98 °C for 10 s, 55 °C for 20 s, 72 °C for 20 s), 72 °C for 5 min. Amplification was verified by running the products on an agarose gel. Indices were added in a subsequent PCR using an Illumina Nextera kit with the following PCR programme: 98 °C for 30 s, 8× (98 °C for 10 s, 55 °C for 20 s, 72 °C for 20 s), 72 °C for 5 min. Attachment of indices was verified by running the products on an agarose gel. Products from the nested PCR were pooled on the basis of band intensity and the resulting library cleaned with magnetic beads. The DNA concentration of pooled libraries was measured fluorometrically. Sequencing was done on an Illumina MiSeq desktop sequencer using the MiSeq reagent kit V3 (Illumina) for 2 × 300 bp paired-end sequencing. Paired-end reads were subsequently trimmed, merged and analysed using the DADA2 (v1.16.0) pipeline^80^.

### Statistical analysis

No data were excluded before the statistical analysis in the present study. No allocation and randomization were included as the study is observational. This study includes all available samples (*n* = 147) of patients with anorexia and healthy individuals. Although no statistical methods were used to predetermine sample sizes, our sample sizes are similar to those reported in previous publications^43,81^. Samples were randomly distributed across metagenomics and metabolomics batches. Investigators were blinded to group allocation during data collection in metagenomic, biochemical and metabolomics analyses. All analyses of human samples were performed using R (v4.1.2). Gene expression analyses and body weight comparisons for animal studies were performed using GraphPad Prism (v9.3.0).

1. Differential analysis
  We carried out the differential analysis using the metadeconfoundR pipeline implemented in the R package metadeconfoundR (v0.1.8; see https://github.com/TillBirkner/ metadeconfoundR or 10.5281/zenodo.4721078) where we assessed the extent to which the observed differences between AN and HC participants in microbiome or metabolome analyses are confounded by covariates including age, BMI, smoking and medication. This pipeline initially used univariate statistics to find associations between microbiome features and disease status, followed by nested linear model comparison post hoc testing to check for the confounding effects of potential covariates and finally, returning a status label.
2. Association analysis
  For the association and mediation analysis between omics features and eating behaviour and psychological traits within the AN group, we first checked the normality of continuous variables with Shapiro-Wilk normality test, finding most variables not to be normally distributed. Therefore, before association analysis, we standardized the continuous variables using empirical normal quantile transformation to follow a standard normal distribution (N ~ (0, 1)). Then we implemented a linear regression model to assess the associations between omics features and eating behaviour and psychological traits using the following formula where confounding factors were added as covariates.
  $$\begin{array}{l}{\mathrm{Eating}}\,{\mathrm{behavior}} - {\mathrm{and}}\,{\mathrm{psychological}}\,{\mathrm{traits}}\sim {\mathrm{omics}}\,{\mathrm{features}} \\ ( {\mathrm{for}}\,{\mathrm{example,}}\, {\mathrm{metabolite/msp/SV}}) \\ + {\mathrm{Age}} + {\mathrm{BMI}} + {\mathrm{Smoking}} + {\mathrm{Medication}}\end{array}$$
  Medication included selective serotonin re-uptake inhibitors, antipsychotics and benzodiazepines.
3. For the association analysis between omics features and host metabolic traits, a normality check and data standardization were also performed before the linear regression analysis. In the linear regression model, the above-mentioned confounding factors were included as covariates, except for BMI as the extremely low BMI is the most remarkable phenotypic change for AN patients compared with HC individuals.

$$\begin{array}{l}{\mathrm{Metabolic}}\,{\mathrm{traits}}( {\mathrm{for}}\,{\mathrm{example,}}\, {\mathrm{BMI/plasma}}\,{\mathrm{glucose}} )\sim {\mathrm{omics}}\,{\mathrm{features}}\\ ( {\mathrm{for}}\,{\mathrm{example,}}\, {\mathrm{metabolite/msp/SV}}) + {\mathrm{Age}} + {\mathrm{Smoking}} + {\mathrm{Medication}}\end{array}$$

Differences in gut microbial diversity (gene richness, species count, taxonomic composition) between AN and HC were calculated using Wilcoxon tests, and Kruskal-Wallis test was used for assessing the significance of differences between multiple groups. Unless otherwise stated, all *P* values were corrected using the Benjamini-Hochberg method and *P*_adj_ < 0.05 was considered statistically significant.

### Reporting summary

Further information on research design is available in the Nature Portfolio Reporting Summary linked to this article.

## Supplementary information

Supplementary Information Supplementary Notes, Figs. 1–7 and References.

Reporting Summary

Supplementary Table Supplementary Tables 1–8.

Supplementary Data Source data for Supplementary Figs. 1–6.

## Data Availability

Anonymized clinical data that are stored in Sharepoint via Odense Patient Data Explorative (file no OP_153) can be accessed by contacting rene.stoeving@rsyd.dk or can be found in Supplementary Table 2. Raw shotgun sequencing data and 16s rRNA gene amplicon sequencing data that support the findings of this study have been deposited in the European Nucleotide Archive with accession numbers PRJEB51776 and PRJEB60103, respectively. Metabolomics data are available from Metabolomics Workbench repository under the link 10.21228/M8KT5B. The KEGG Database is available at https://www.genome.jp/kegg/. Source data are provided with this paper.

## Source data

Source Data Fig. 1 Statistical source data.
Source Data Fig. 2 Statistical source data.
Source Data Fig. 3 Statistical source data.
Source Data Fig. 4 Statistical source data.
Source Data Fig. 5 Statistical source data.
Source Data Fig. 6 Statistical source data.
Source Data Extended Data Fig. 2 Statistical source data.
Source Data Extended Data Fig. 4 Statistical source data.
Source Data Extended Data Fig. 5 Statistical source data.
Source Data Extended Data Fig. 6 Statistical source data.
Source Data Extended Data Fig. 7 Statistical source data.
Source Data Extended Data Fig. 8 Statistical source data.
Source Data Extended Data Fig. 10 Statistical source data.

## Code availability

Codes associated with the data analysis and visualization are available at https://github.com/fjw536/AnorexiaGutMicrobiome.git.
