# Supplementary material for: The gut microbiota contributes to the pathogenesis of anorexia nervosa in humans and mice
Source: Nat Microbiol. 2023 Apr 17;8(5):787–802. doi: 10.1038/s41564-023-01355-5 (PMC10159860; doi:10.1038/s41564-023-01355-5)
Supplement: Supplementary file 1 — Supplementary Notes, Figs. 1–7 and References. [file 41564_2023_1355_MOESM1_ESM.pdf]

nature microbiology

Article https://doi.org/10.1038/s41564-023-01355-5

# The gut microbiota contributes to the pathogenesis of anorexia nervosa in humans and mice

In the format provided by the authors and unedited

## Contents

**Supplementary Notes:** Supplementary introductions, results, and discussions

**Supplementary Figure 1:** Boxplot at species level of β-diversity in the gut microbiota between inpatient and outpatient AN individuals

**Supplementary Figure 2:** Heatmaps showing associations between bacterial taxa and bioclinical variables in all 147 study participants (77 anorexia nervosa cases and 70 healthy controls)

**Supplementary Figure 3:** Heatmap showing transkingdom ecologic correlations between the viral and bacterial gut microbiota in normal control (left panel) compared with anorexia nervosa (right panel) patients

**Supplementary Figure 4:** Heatmap showing the transkingdom ecologic correlations between the viral and bacterial gut microbiota in binge-purge anorexia nervosa cases (left) and restrictive anorexia nervosa cases (right)

**Supplementary Figure 5:** Alterations in viral gut microbiota of AN cases and its subtypes

**Supplementary Figure 6:** Distribution of structural variants in AN bacteriome

**Supplementary Figure 7:** Workflow of bacterial cell counting in human fecal samples

**Supplementary References**

## Supplementary Notes: Supplementary introductions, results, and discussions

### Supplementary Note 1: Introduction of previous reports on gut microbiome studies in AN

Several small studies that used amplicon sequencing to characterize the gut microbiota at the genus level in AN have reported dysbiosis of gut bacterial microbiota. For example, a PCR-based study on the fecal microbiota of nine French female AN cases and 20 control subjects[81] suggested a higher abundance of methanogen species in AN cases. In a study of 25 Japanese females with AN and 21 age-matched healthy females, using 16S- or 23S rRNA-gene marker approaches, a significant difference in the abundance of anaerobic and facultative aerobic bacteria was reported[82]. Lower bacterial diversity was reported in a US study, which used 16S rRNA gene sequencing of stools from 16 hospitalized females with AN and 12 controls[83]. Finally, in a German gut microbiota study comprising 55 females with AN and 55 controls, 16S rRNA gene sequencing revealed a higher abundance of mucin-degraders and members of *Clostridium* clusters I, XI and XVIII, and lower abundance of butyrate-producing *Roseburia spp.*[84].

### Supplementary Note 2: Description of Eating Disorder Inventory (EDI-3)

EDI-3 consists of 91 items organized onto 12 primary scales, consisting of three eating-disorder-specific scales (Drive for Thinness, Bulimina and Body Dissatisfaction) and nine general psychological scales that are highly relevant to, but not specific to, eating disorders (see below)[85]. EDI-3 developed from current theories related to pathogenesis and diagnosis of eating disorders and it was designed for use in females aged 13–53 years. The inventory is owned by Psychological Assessment Resources: www.parinc.com. EDI-3 was clinically validated in the Danish health care system[85].

Short description of the 12 subscales of EDI-3 is given below:

*Drive for Thinness (DT)*

The "Drive for Thinness" is considered as one of the cardinal features of eating disorders and has been considered an essential criterion for a diagnosis of these abnormalities. The seven items on this scale assess an extreme desire to be thinner, concern with dieting, preoccupation with body weight and an intense fear of weight gain. Prospective studies have indicated that the DT scale is a predictor of binge-eating and the development of regular eating disorders.

*Bulimia (B)*

The Bulimia construct assesses the tendency to think about and to engage in bouts of uncontrollable overeating (binge-eating). The eight items on this scale assess concerns about overeating and eating in

response to being upset. The presence of binge eating is one of the defining features of bulimia nervosa and differentiates the bingeing/purging and restrictive subtypes of anorexia nervosa. Binge eating is common in individuals who do not meet all of the criteria to qualify for a formal diagnosis of an eating disorder; however, in most cases, severe binge eating is associated with marked psychological distress.

*Body Dissatisfaction (BD)*

The Body Dissatisfaction scale consists of 10 items that assess discontentment with the overall shape and with the size of regions of the body of extraordinary concern to those with eating disorders (i.e., stomach, hips, thighs, buttocks). One item on BD scale measures the feeling of bloating after eating a normal meal, a common feature of those who are dissatisfied with their body weight. Given the fact that body dissatisfaction is endemic to young women in Western culture, it is does not relate to any disorder on its own; however, BD is considered to be a major risk factor responsible for initiating and then sustaining extreme weight controlling behaviors seen in individuals with eating disorders.

*Low Self-Esteem (LSE)*

The Low Self-Esteem (LSE) construct measures basic concept of negative self-evaluation. Five of the six items of LSE are assessing feelings of insecurity, inadequacy, ineffectiveness, and lack of personal worth. The remaining item is an estimate of self-perception of being unable to achieve personal standards. A low SLE plays a major role in the development and maintenance of eating disorders.

*Personal Alienation (PA)*

The Personal Alienation (PA) construct is conceptually related to low self-esteem; however, this construct also reflects a pervasive sense of emotional emptiness, aloneness and poor sense of self-understanding. The seven items in PA include content reflecting feelings of being separated from others and losing out or not being given due credit from others. PA items also measure the wish to be someone else and a general sense of being out of control of things in personal life.

*Interpersonal Insecurity (II)*

The Interpersonal Insecurity (II) scale consists of seven items assessing discomfort apprehension, and reticence in social situations. The II scale focuses particularly on difficulties expressing personal thoughts and feelings with others. Item content on the II scale also assess the tendency to withdrawal and isolate from others.

*Interpersonal Alienation (IA)*

The Interpersonal Alienation (IA) scale includes seven items that evaluate disappointment, distance, estrangement, and lack of trust in relationships. Item content also measures the tendency to feel trapped in relationships as well as the sense that there is a lack of understanding and love from others. A high score on the IA scale indicates a basic impairment of attachment in relationships.

*Interoceptive Deficits (ID)*

The Interoceptive Deficits (ID) scale consists of nine items that estimate confusion related to accurately recognizing and responding to emotional states. There is a "fear of affect" item cluster indicating distress when emotions are too strong or out of control that contrasts with an "affective confusion" item cluster indicating difficulty in accurately recognize emotional states. Confusion and mistrust related to affective and bodily functioning have been repeatedly described as an important characteristic of those who develop eating disorders.

*Emotional Dysregulation (ED)*

The Emotional Dysregulation (ID) scale consists of eight items assessing a tendency toward mood instability, impulsivity, recklessness, anger, and self-destructiveness. There are two items indicating potential problems with substance abuse; one for alcohol and one for drugs. The tendency toward poor emotion regulation and mood intolerance has been identified as a poor prognostic sign in eating disorders.

*Perfectionism (P)*

The Perfectionism (P) scale consists of six items evaluating the extent to which a person places a premium on achieving a high goal and standard of personal achievement. Item content on the Perfectionism scale falls into two clusters: Three items measure "personal perfectionistic standards" reflecting demanding personal standards for performance and three items assessing "parental perfectionistic standards" indicating pressures from parents and teachers. Perfectionism is considered at the heart of relentless efforts of weight control as well as unrealistic strivings in other areas. It has been identified as a key feature in the development and maintenance of eating disorders.

*Ascetism (A)*

The Asceticism (A) scale consists of seven items assessing the tendency to seek virtue through the pursuit of spiritual ideals such as self-discipline, self-denial, self-restraint, self-sacrifice and control of bodily urges. A 3-item "suffering" cluster relates to the concept that self-denial makes a person stronger; a "weakness" cluster measures the tendency to view pleasure, relaxing and human weakness as shameful.

*Maturity Fears (MF)*

The Maturity Fears (MF) scale consists of eight items assessing the desire to retreat to the security of childhood. This construct has been described as a central maintaining feature in a subgroup of adolescent individuals, whose dieting and weight loss is functional in that it provides a means to return to a pre-pubertal appearance and hormonal status that allows them to retreat from turmoil, conflicts and developmental expectations associated with adulthood.

## Supplementary Note 3: Gut bacterial genus and species associated with bioclinical variables in the combined AN and HC cohort

At the genus level, we found a positive correlation between *Bifidobacterium* absolute abundance and body mass index (BMI). In contrast, *Lactobacillus,* which was more abundant in AN, was inversely correlated with BMI. *Roseburia* had a higher absolute abundance in HC, which correlated with BMI and plasma C reactive protein levels (Supplementary Figure 2a). In an analysis of all 147 study participants, alterations in AN-associated bacterial species correlated with seven bioclinical variables, including BMI and circulating concentrations of C-reactive protein, creatinine, low-density lipoprotein cholesterol (LDL-C), insulin, glucose, and HOMA-IR (Supplementary Figure 2b).

**Supplementary Note 4: ClpB measurement and discussion on the role of ClpB in AN**

We observed no significant difference between groups in the absolute abundance of *Enterobacteriaceae*, a family with the potential to synthesize anorexigenic ClpB; a conformational mimetic of α-melanocyte-stimulating hormone (α-MSH)[86] ($P_{Wilcoxon} = 0.28$; Extended Data Figure 5a). Moreover, we did not confirm the findings of Breton et al.[86] of higher circulating levels of ClpB in AN ($P_{Wilcoxon} = 0.31$; Extended Data Figure 5b), which may be due to the inclusion of both AN cases and obese subjects with binge eating disorder. In both studies the inter-individual variation in plasma levels of ClpB was high. Here, we observed a higher level of ClpB concentrations in HC compared to AN-BP ($P_{Wilcoxon} = 9.2e\text{-}02$; Extended Data Figure 5c).

We did not confirm a higher plasma level of ClpB in AN. While the increased ClpB plasma level in AN-RS is pleading for the previously described activation of satiety by ClpB[86], the role of ClpB in AN pathology remains unclear. It is of interest, however, that investigators of a recent double blind placebo-controlled clinical trial reported that supplementation with a ClpB producing probiotic bacterial strain under hypocaloric dieting in overweight subjects improved weight loss[87].

**Supplementary Note 5: Bacterial genetics link to metabolism-related bioclinical variables**

In bacterial genetics analyses, we identified the presence of a 1-kbp variable SV in the *Alistipes putredinis* genome that was associated with higher insulin sensitivity and lower circulating concentrations of glucose and insulin across the whole cohort (Extended Data Figure 7a-7c). Within the region of this SV is a gene encoding a SMI1/KNR4 family (SUKH-1) protein that is involved in up-regulation of 1,3-beta-glucan synthase activity[88] (Extended Data Figure 7d). This

has previously been linked to improvements in insulin resistance, dyslipidemia, hypertension, and obesity[89].

**Supplementary Note 6: Dysregulation of valine and fatty acid metabolism in AN**

We also observed a depletion in serum levels of valine in AN, which has been linked to higher insulin sensitivity[90]. Serum concentrations of saturated fatty acid C10:0, long-chain unsaturated fatty acids including C16:1 and C18:3, and a very long-chain unsaturated fatty acid, that is C22:6, were lower in AN (Figure 5b). This may be a result of dietary restrictions of essential and poly-unsaturated fatty acids in AN[91].

**Supplementary Note 7: Discussion on the mechanism behind the gradual body weight gain in both AN-T and HC-T mice**

The mechanisms behind the gradual gain of weight over time in both groups of mice after the initial weight loss despite continued energy restriction are unknown but might be related to a metabolic adaptation including changes in physical activity and thereby energy expenditure[92].

**Supplementary Figures**

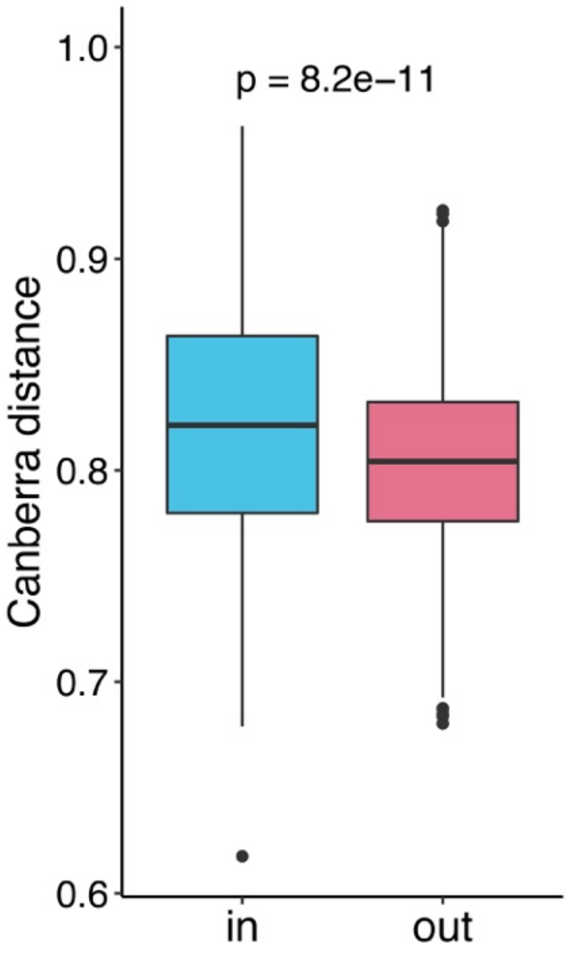


**Supplementary Figure 1. β-diversity at bacterial species level (Canberra distance) in the gut microbiota between in- and out-AN cases with anorexia nervosa.** Box plots show the median with hinges that correspond to the 25th and 75th percentiles. The whiskers extend from the hinge to the largest and smallest value no further than 1.5 multiplied by the inter-quartile range. Two-tailed Wilcoxon rank-sum test was used to determine significance. In denotes inpatients ($n$ = 35); out denotes outpatients ($n$ = 42).

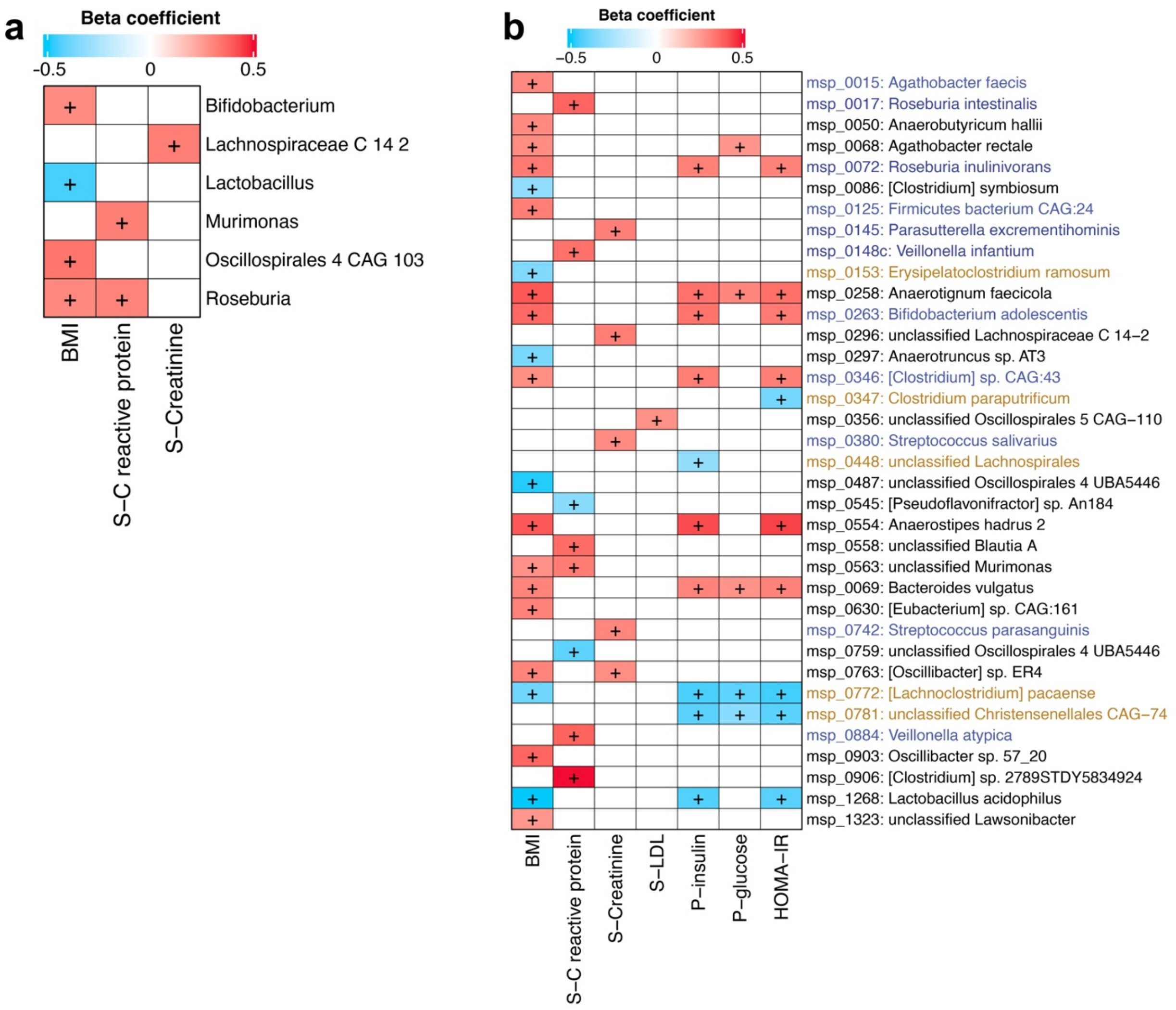


**Supplementary Figure 2. Heatmaps showing associations between bacterial taxa and bioclinical variables in all 147 study participants.** **a**,**b**, Heatmaps of the association between clinical variables and abundance of gut bacteria at (**a**) genus, and (**b**) species level after deconfounding for multiple covariates including age, smoking, and medication. In **b**, AN-enriched MSPs are marked in gold, and HC-enriched MSPs are in blue. +, adjusted p-value < 0.1 after Benjamini-Hochberg correction (see Source Data for exact p values).

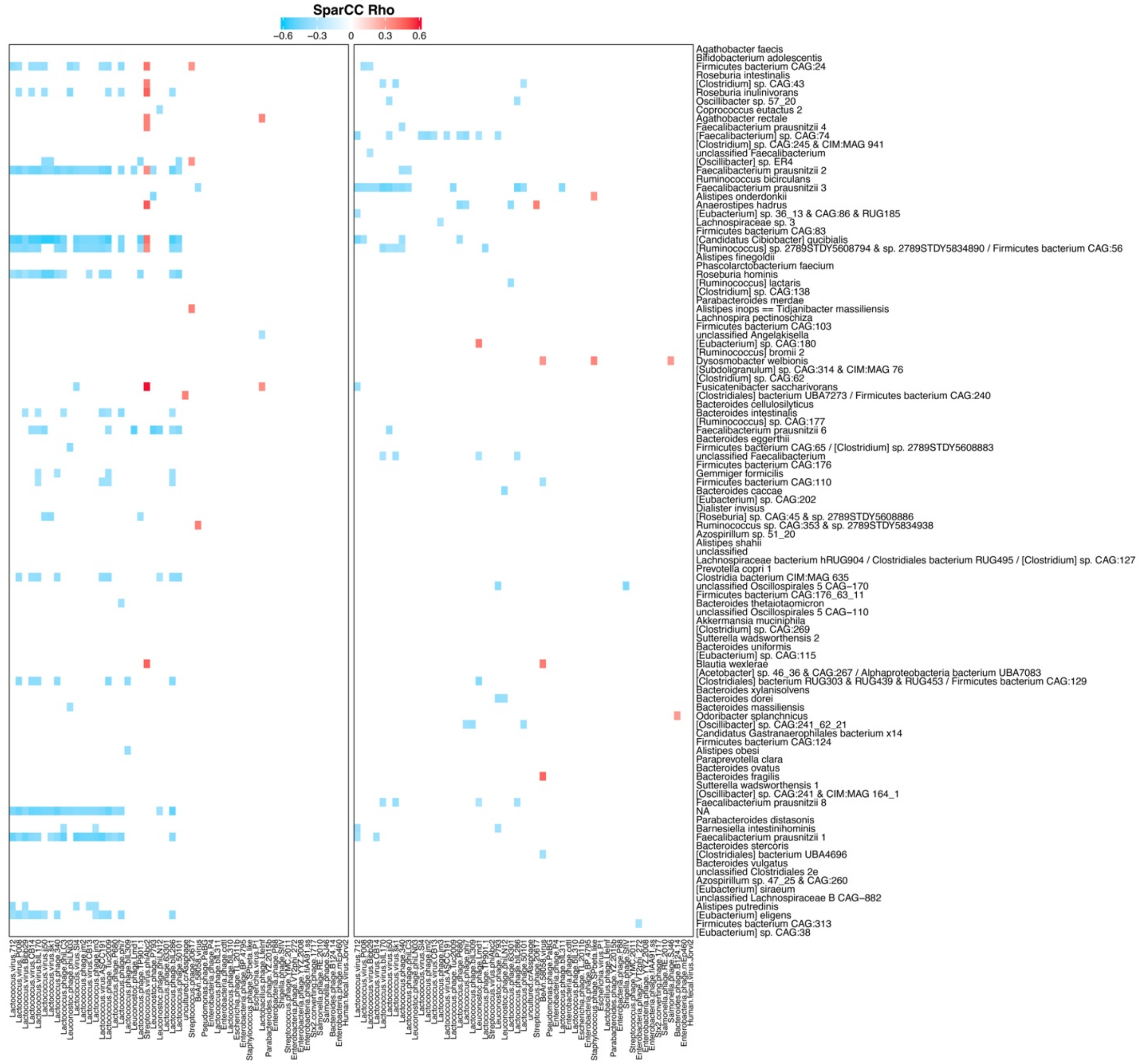


**Supplementary Figure 3. Heatmap showing the transkingdom ecologic correlations between the viral and bacterial gut microbiota in healthy control subjects (left panel) compared with anorexia nervosa (right panel) patients.** Correlation coefficients were computed and corrected for compositional effects using the SparCC algorithm. Bacterial or viral taxa with relative abundance > 0.01% were selected for Sparse Correlations for Compositional (SparCC) calculation. Correlations with coefficient values of > 0.4 or ≤ 0.4 and adjusted p-value < 0.05 were defined as significant and visualized.

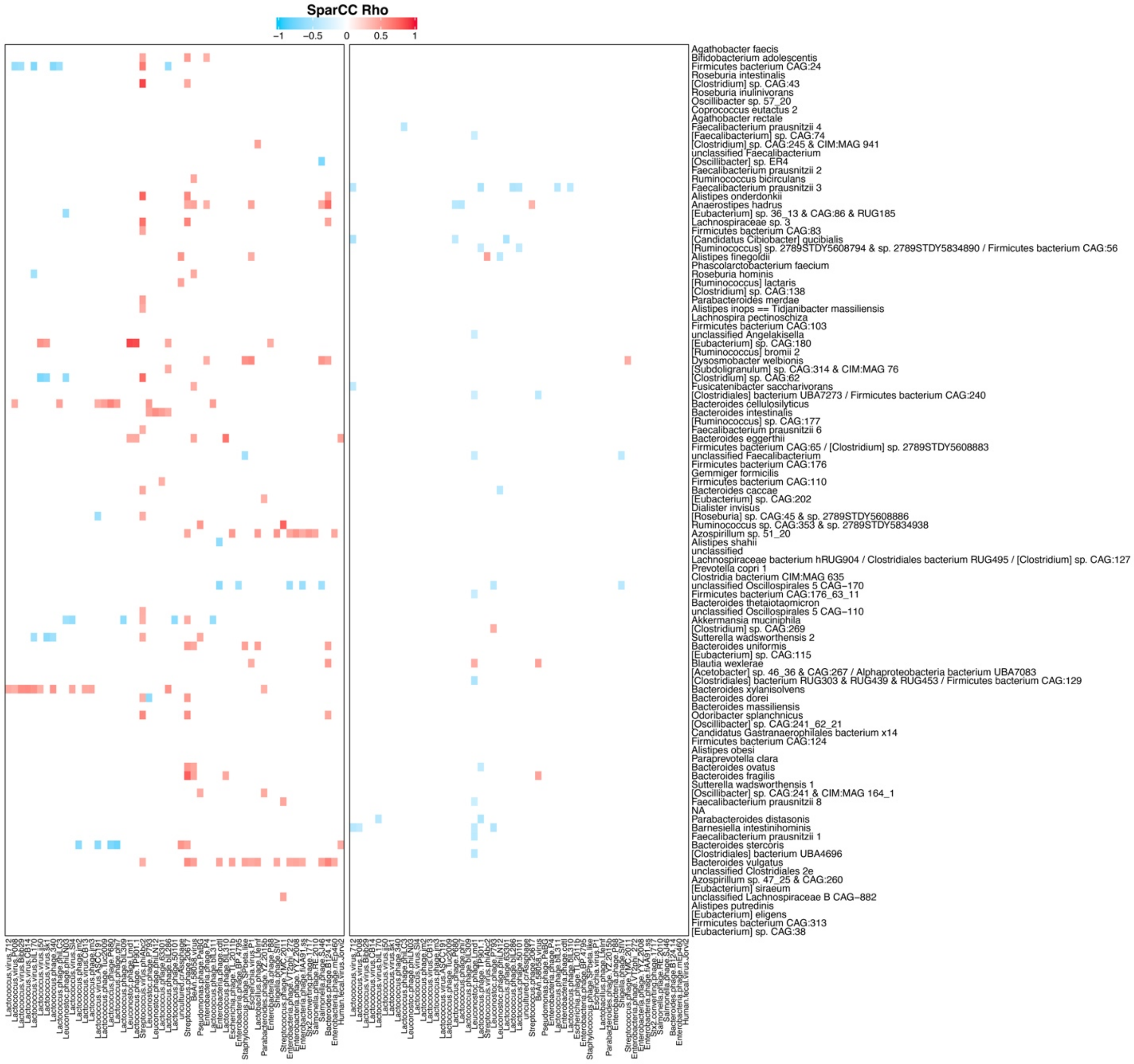


**Supplementary Figure 4. Heatmap showing the transkingdom ecologic correlations between the viral and bacterial gut microbiota in binge-purge anorexia nervosa cases (left) and restrictive anorexia nervosa cases (right).** Correlation coefficients were computed and corrected for compositional effects using the SparCC algorithm. Bacterial or viral taxa with relative abundance > 0.01% were selected for Sparse Correlations for Compositional (SparCC) calculation. Correlations with coefficient values of > 0.4 or ≤ 0.4 and adjusted p-value < 0.05 were defined as significant and visualized.

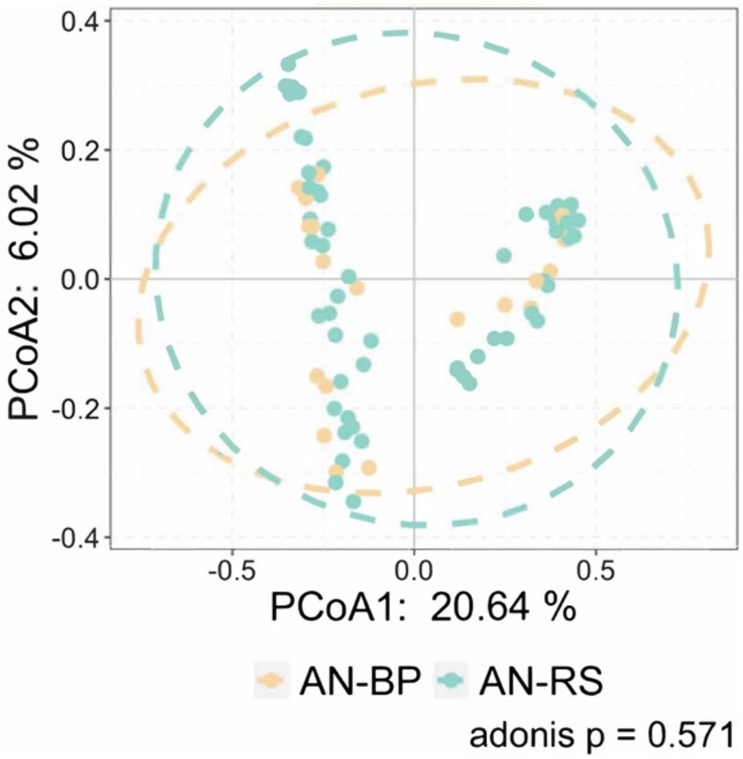


**Supplementary Figure 5. Alterations in viral gut microbiota of AN cases and its subtypes.** Principal Coordinates Analysis (PCoA) of the Canberra distance showing the stratification of AN from NC in the gut virome at species level. Statistical significance for the Canberra distance was tested by PERMANOVA with permutations done 999 times. AN-BP, binge/purge AN (*n* = 21); AN-RS, restrictive AN (*n* = 56).

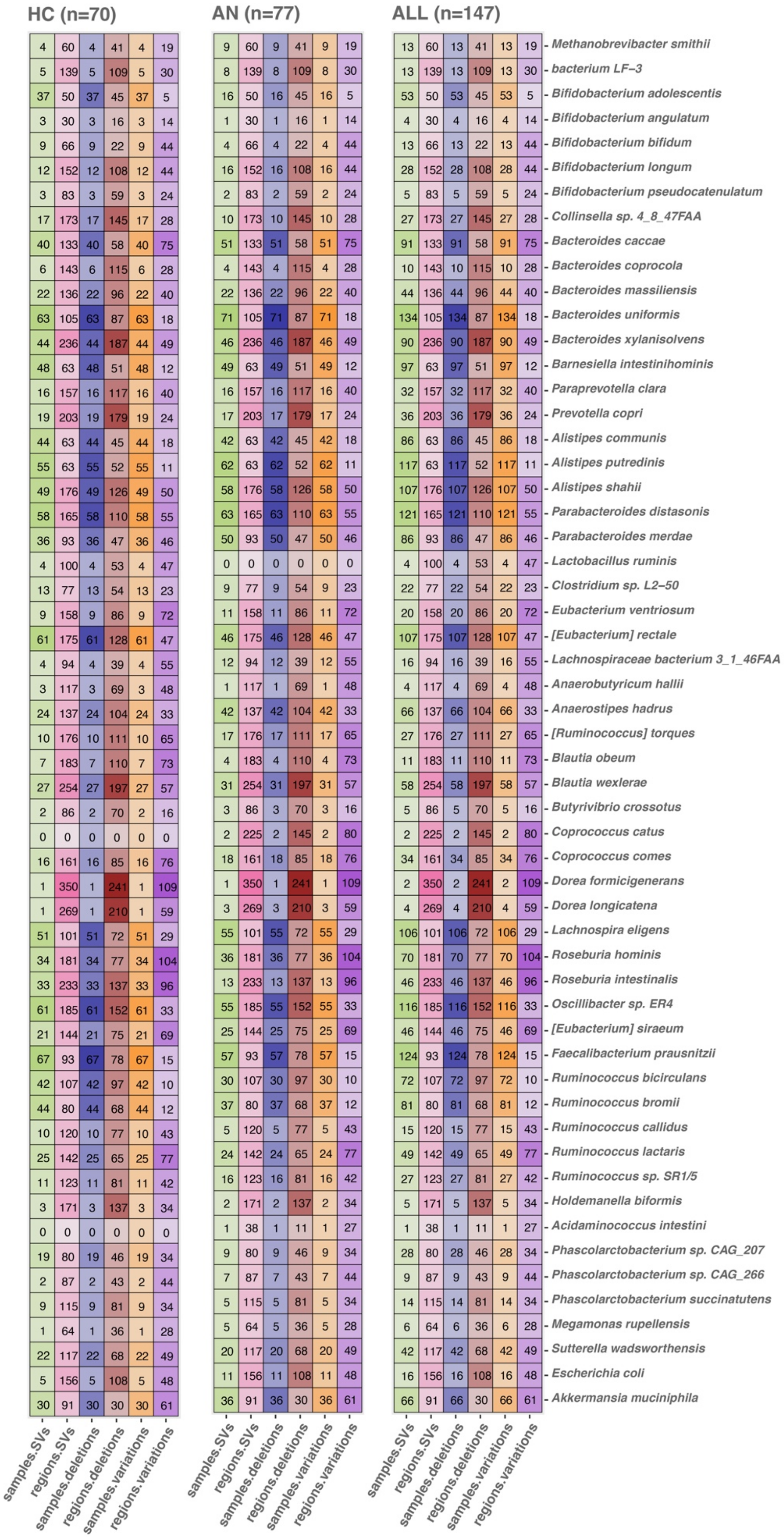
HC (n=70)
AN (n=77)
ALL (n=147)
Methanobrevibacter smithii
bacterium LF-3
Bifidobacterium adolescentis
Bifidobacterium angulatum
Bifidobacterium bifidum
Bifidobacterium longum
Bifidobacterium pseudocatenulatum
Collinsella sp. 4_8_47FAA
Bacteroides caccae
Bacteroides coprocola
Bacteroides massiliensis
Bacteroides uniformis
Bacteroides xylanisolvens
Barnesiella intestinihominis
Paraprevotella clara
Prevotella copri
Alistipes communis
Alistipes putredinis
Alistipes shahii
Parabacteroides distasonis
Parabacteroides merdae
Lactobacillus ruminis
Clostridium sp. L2-50
Eubacterium ventriosum
[Eubacterium] rectale
Lachnospiraceae bacterium 3_1_46FAA
Anaerobutyricum hallii
Anaerostipes hadrus
[Ruminococcus] torques
Blautia obeum
Blautia wexlerae
Butyrivibrio crossotus
Coprococcus catus
Coprococcus comes
Dorea formicigenerans
Dorea longicatena
Lachnospira eligens
Roseburia hominis
Roseburia intestinalis
Oscillibacter sp. ER4
[Eubacterium] siraeum
Faecalibacterium prausnitzii
Ruminococcus bicirculans
Ruminococcus bromii
Ruminococcus callidus
Ruminococcus lactaris
Ruminococcus sp. SR1/5
Holdemanella biformis
Acidaminococcus intestini
Phascolarctobacterium sp. CAG_207
Phascolarctobacterium sp. CAG_266
Phascolarctobacterium succinatutens
Megamonas rupellensis
Sutterella wadsworthensis
Escherichia coli
Akkermansia muciniphila
samples.SVs
regions.SVs
samples.deletions
regions.deletions
samples.variations
regions.variations

**Supplementary Figure 6. Distribution of structural variants (SVs) in AN bacteriome.** In each panel, from left to right, the columns represent: number of samples that present SVs per bacterium, the total number of SVs regions per bacterium, the number of samples with deletions per bacterium, the number of distinct deleted regions per bacterium, the number of samples with variable SVs per bacterium and the number of variable SVs regions detected per bacterium. Transparency of the cells color represent the number of either samples or SVs per bacterium, being the most transparent the minimum samples and the less transparent the maximum.

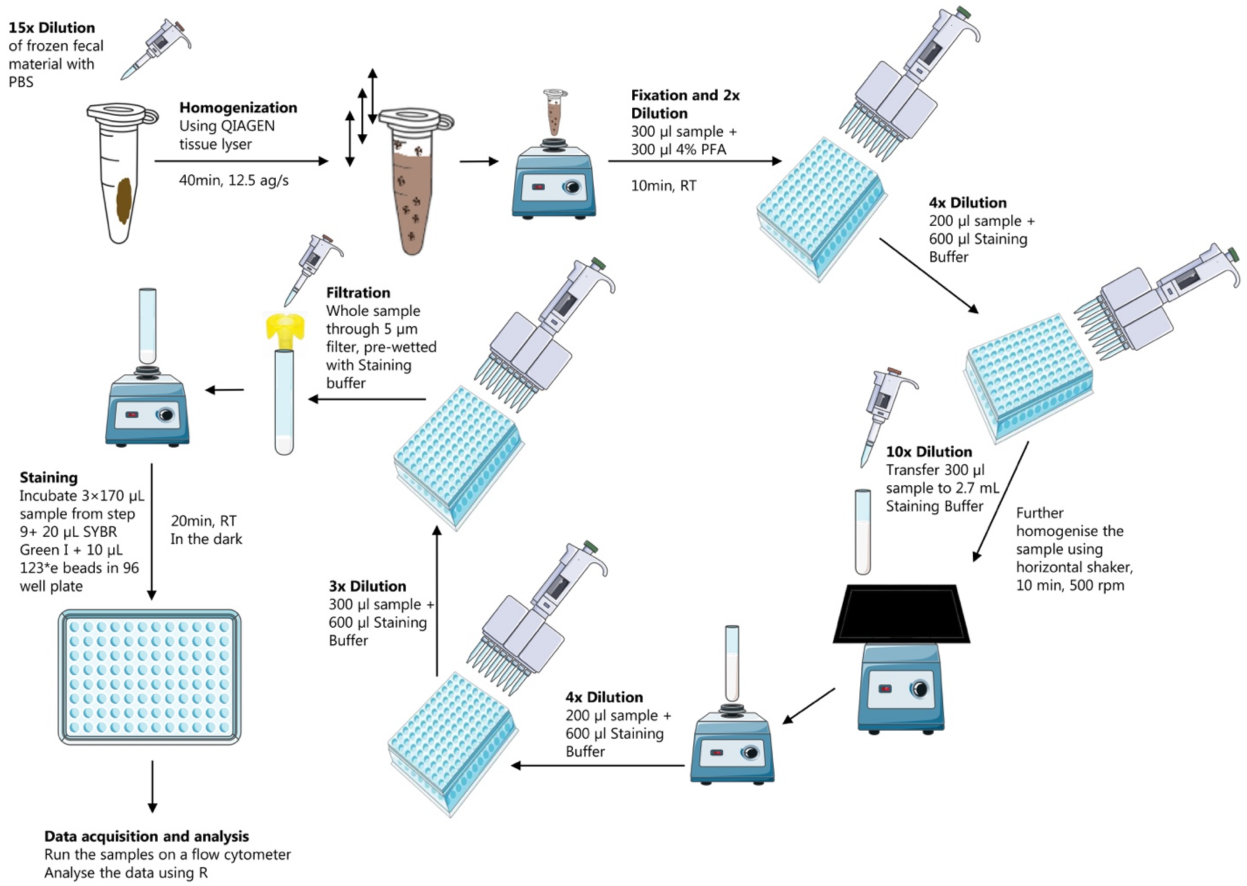


**Supplementary Figure 7. Workflow of bacterial cell counting procedure in human stool sample.** PBS, phosphate-buffered saline; PFA, paraformaldehyde; RT, room temperature; rpm, rotation per minute. The Figure was generated using Servier Medical Art, provided by Servier, licensed under a Creative Commons Attribution 3.0 unported license.

## Supplementary References


81 Armougom, F., Henry, M., Vialettes, B., Raccah, D. & Raoult, D. Monitoring bacterial community of human gut microbiota reveals an increase in Lactobacillus in obese patients and Methanogens in anorexic patients. *PLoS ONE* **4**, e7125 (2009).

82 Morita, C. *et al.* Gut dysbiosis in patients with anorexia nervosa. *PLoS ONE* **10**, e0145274 (2015).

83 Kleiman, S. C. *et al.* The intestinal microbiota in acute anorexia nervosa and during renourishment: relationship to depression, anxiety, and eating disorder psychopathology. *Psychosom. Med.* **77**, 969 (2015).

84 Mack, I. *et al.* Weight gain in anorexia nervosa does not ameliorate the faecal microbiota, branched chain fatty acid profiles and gastrointestinal complaints. *Sci. Rep.* **6**, 1-16 (2016).

85 Clausen, L., Rosenvinge, J. H., Friborg, O. & Rokkedal, K. Validating the Eating Disorder Inventory-3 (EDI-3): A comparison between 561 female eating disorders patients and 878 females from the general population. *J. Psychopathol. Behav. Assess.* **33**, 101-110 (2011).

86 Breton, J. *et al.* Elevated plasma concentrations of bacterial ClpB protein in patients with eating disorders. *Int. J. Eating Disord.* **49**, 805-808 (2016).

87 Déchelotte, P. *et al.* The probiotic strain H. alvei HA4597® improves weight loss in overweight subjects under moderate hypocaloric diet: A proof-of-concept, multicenter randomized, double-blind placebo-controlled study. *Nutrients* **13**, 1902 (2021).

88 Hong, Z. *et al.* Cloning and characterization of KNR4, a yeast gene involved in (1, 3)-beta-glucan synthesis. *Mol. Cell. Biol.* **14**, 1017-1025 (1994).

89 El Khoury, D., Cuda, C., Luhovyy, B. & Anderson, G. Beta glucan: health benefits in obesity and metabolic syndrome. *J. Nutr. Metab.* **2012** (2012).

90 Pedersen, H. K. *et al.* Human gut microbes impact host serum metabolome and insulin sensitivity. *Nature* **535**, 376-381 (2016).

91 Shimizu, M. *et al.* Very long chain fatty acids are an important marker of nutritional status in patients with anorexia nervosa: a case control study. *Biopsychosoc. Med.* **14**, 1-9 (2020).

92 Basolo, A., Magno, S., Santini, F. & Ceccarini, G. Ketogenic Diet and Weight Loss: Is There an Effect on Energy Expenditure? *Nutrients* **14**, 1814 (2022).
