# Supplementary material for: The gut microbiota contributes to the pathogenesis of anorexia nervosa in humans and mice
Source: Nat Microbiol. 2023 Apr 17;8(5):787–802. doi: 10.1038/s41564-023-01355-5 (PMC10159860; doi:10.1038/s41564-023-01355-5)
Supplement: Supplementary file 2 — Reporting Summary [file 41564_2023_1355_MOESM2_ESM.pdf]

nature portfolio

Corresponding author(s): Oluf Pedersen

Last updated by author(s): Mar 2, 2023

Reporting Summary

Nature Portfolio wishes to improve the reproducibility of the work that we publish. This form provides structure for consistency and transparency in reporting. For further information on Nature Portfolio policies, see our Editorial Policies and the Editorial Policy Checklist.

Statistics

For all statistical analyses, confirm that the following items are present in the figure legend, table legend, main text, or Methods section.

n/a	Confirmed	
☐	☒	The exact sample size (*n*) for each experimental group/condition, given as a discrete number and unit of measurement
☐	☒	A statement on whether measurements were taken from distinct samples or whether the same sample was measured repeatedly
☐	☒	The statistical test(s) used AND whether they are one- or two-sided<br>*Only common tests should be described solely by name; describe more complex techniques in the Methods section.*
☒	☐	A description of all covariates tested
☐	☒	A description of any assumptions or corrections, such as tests of normality and adjustment for multiple comparisons
☐	☒	A full description of the statistical parameters including central tendency (e.g. means) or other basic estimates (e.g. regression coefficient) AND variation (e.g. standard deviation) or associated estimates of uncertainty (e.g. confidence intervals)
☐	☒	For null hypothesis testing, the test statistic (e.g. *F*, *t*, *r*) with confidence intervals, effect sizes, degrees of freedom and *P* value noted<br>*Give P values as exact values whenever suitable.*
☒	☐	For Bayesian analysis, information on the choice of priors and Markov chain Monte Carlo settings
☒	☐	For hierarchical and complex designs, identification of the appropriate level for tests and full reporting of outcomes
☐	☒	Estimates of effect sizes (e.g. Cohen's *d*, Pearson's *r*), indicating how they were calculated

Our web collection on statistics for biologists contains articles on many of the points above.

Software and code

Policy information about availability of computer code

Data collection	See manuscript and Online Methods for details;<br>Bacterial cell count was measured using a BD Fortessa LSRII flow cytometer (BD Biosciences) and data were acquired using BD FACSDiVaTM software and processed using R package folowcore (v1.11.20). Metagenomic sequencing was performed on the Ion Proton Sequencer (ThermoFisher Scientific, Waltham, US) system, with a minimum of 20 million high-quality reads of 150 bp (in average) generated per library. METEOR v3.2 (https://forgemia.inra.fr/metagenopolis/meteor) was used to construct the gene count table of microbiome data; low-quality reads were filtered by AlienTrimmer (v0.4.0); Bowtie2 (v2.3.4) was used for reads alignment to Integrated Gut Catalog 2; R package MetaOMineR (v1.31) was used to generate the down-sized gene matrix; gut metabolic modules were generated by R package omixerRpm (v 0.3.2); in studies of structural variations (SVs) of gut bacterial genomes we used quality filtered FastQ files to compute the abundance and prevalence of copy number variations and deletions with SGVFinder software (https://github.com/segalab/SGVFinder), applying default parameters. Viral gut microbiota was profiled by MiCoP (https://github.com/smangul1/MiCoP). MassHunter B.06.01 software (Agilent Technologies, Santa Clara, CA, USA) was used for all metabolomics data acquisition.<br>16S rRNA sequencing of stool samples obtained from human donors and mouse recipients was done on an Illumina MiSeq desktop sequencer using the MiSeq Reagent Kit V3 (Illumina) for 2x 300 bp paired-end sequencing. Paired-end reads were subsequently trimed, merged, and analysed using DADA2 package (1.16.0) under R environment (v4.1.2).
Data analysis	Most statistical analysis was conducted using the R (v4.1.2) as described in Methods and Online Methods. In particular the package metadeconfoundR (v0.1.8 - see https://github.com/TillBirkner/metadeconfoundR or https://doi.org/10.5281/zenodo.4721078) was employed for analyzing multi-omics data of human. The software Prism (9.3.0) was used in data analysis of animal studies. In addition we applied custom R scripts (see Code Availability or https://github.com/fjw536/AnorexiaGutMicrobiome).

For manuscripts utilizing custom algorithms or software that are central to the research but not yet described in published literature, software must be made available to editors and reviewers. We strongly encourage code deposition in a community repository (e.g. GitHub). See the Nature Portfolio guidelines for submitting code & software for further information.

Data

Policy information about availability of data

All manuscripts must include a data availability statement. This statement should provide the following information, where applicable:

- Accession codes, unique identifiers, or web links for publicly available datasets
- A description of any restrictions on data availability
- For clinical datasets or third party data, please ensure that the statement adheres to our policy

Anonymized clinical data that are stored in Sharepoint via Odense Patient Data Explorative (file no OP_153) can be accessed through contacting rene.stoeving@rsyd.dk or can be found in Supplementary Table 2. Raw shotgun sequencing data and 16s rRNA gene amplicon sequencing data that support the findings of this study have been deposited in the European Nucleotide Archive with accession number PRJEB51776, and PRJEB60103, respectively. Metabolome data has been uploaded to Metabolomics Workbench under project PR001610 with track IDs ST002494 (polar metabolites), and ST002495 (bile acids). The KEGG Database is available at https://www.genome.jp/kegg/.

Human research participants

Policy information about studies involving human research participants and Sex and Gender in Research.

Reporting on sex and gender	As anorexia nervosa occurs in about 95% of cases in women, the participants of this study are all females.
Population characteristics	This is described in detail within the manuscript as part of what we study, relative to both geographical, clinical, demographic and treatment factors. Briefly, 77 women (age 23.5 ± 7.0 years) with anorexia and 70 healthy control women (age 23.2 ± 4.4 years) were enrolled in this study. As expected, anorexia women have low body mass index (BMI, 15.6 ± 2.5 kg/m2), and BMI of healthy control women is 21.8 ± 1.9 kg/m2.
Recruitment	Since 90 - 95% of individuals with diagnosed anorexia (AN) are females, we decided to include only women in the present study and since ethnicity may influence gut microbiota, we only included Danish Caucasian women with AN cases recruited from three specialized centers in Denmark.Exclusion criteria comprised antibiotic, or antifungal treatment within the previous three months, any acute or chronic somatic diseases or infections. All the included patients were treated in specialized centers, and they were interviewed by an experienced and specialized psychologist or psychiatrist at the start of their treatment. The validated Eating Disorder Inventory (EDI, details given in Supplementary Note) was used for interview and as a questionnaire filled out by trained health professional specialists. The exclusion criteria for the age-matched healthy control woman were BMI below 18.5 or above 25, regular medication of any kind apart from birth control pills, and antibiotics within the last three months. The control subjects were recruited via public advertisement and via direct contact to health staff, medical students, and their relatives.
Ethics oversight	The study protocol is registered at ClinicalTrials.gov (NCT02217384) and the study is approved by The Regional Scientific Ethical Committee for Southern Denmark (file no 42053 S-20140040), and all participants involved in this study provided written informed consent.

Note that full information on the approval of the study protocol must also be provided in the manuscript.

Field-specific reporting

Please select the one below that is the best fit for your research. If you are not sure, read the appropriate sections before making your selection.

☒ Life sciences ☐ Behavioural & social sciences ☐ Ecological, evolutionary & environmental sciences

For a reference copy of the document with all sections, see nature.com/documents/nr-reporting-summary-flat.pdf

Life sciences study design

All studies must disclose on these points even when the disclosure is negative.

Sample size	This is an exploratory study and no sample size calculations were performed.
Data exclusions	No data was excluded.
Replication	As a hypothesis generating study, no explicit replication attempts were made. As for the mouse study, the fecal microbiota transplantation was performed in 3 independent batches of germ-free mice. All attempts at replication were successful and supported the conclusions in the manuscript.
Randomization	As the study was observational, there was no allocation or randomization. Samples for metagenomics and metabolomics profilings were randomly distributed across batches.

Blinding	Investigators were blinded to the group allocation during data collection in metagenomic, biochemical, and metabolomics analyses.

Reporting for specific materials, systems and methods

We require information from authors about some types of materials, experimental systems and methods used in many studies. Here, indicate whether each material, system or method listed is relevant to your study. If you are not sure if a list item applies to your research, read the appropriate section before selecting a response.

Materials & experimental systems

n/a	Involved in the study
☒	☐ Antibodies
☒	☐ Eukaryotic cell lines
☒	☐ Palaeontology and archaeology
☐	☒ Animals and other organisms
☐	☒ Clinical data
☒	☐ Dual use research of concern

Methods

n/a	Involved in the study
☒	☐ ChIP-seq
☒	☐ Flow cytometry
☒	☐ MRI-based neuroimaging

Animals and other research organisms

Policy information about studies involving animals; ARRIVE guidelines recommended for reporting animal research, and Sex and Gender in Research

Laboratory animals	Germ-free Swiss Webster mice (6-week old, female) was used for the fecal microbiota transplantation.
Wild animals	No wild animals were used.
Reporting on sex	Female mice were used.
Field-collected samples	This work does not utilize field-collected samples.
Ethics oversight	Animal protocols were approved by The Science Ethics Committees for the Capital Region of Copenhagen, Denmark.

Note that full information on the approval of the study protocol must also be provided in the manuscript.

Clinical data

Policy information about clinical studies

All manuscripts should comply with the ICMJE guidelines for publication of clinical research and a completed CONSORT checklist must be included with all submissions.

Clinical trial registration	The study protocol is registered at ClinicalTrials.gov (NCT02217384).
Study protocol	Available from the corresponding author.
Data collection	This is described in greater detail in the manuscript, but involve hospital regions from three specialized centers in Denmark. The centers were Center for Eating Disorders, Odense University Hospital, Child and Adolescent Psychiatric Unit, Aarhus University Hospital, and Unit for Psychiatric Research, Aalborg University Hospital. The recruitment and data collection took place from 2014 September 1, 2014 to July 31, 2016.
Outcomes	No outcomes were tracked; cross-sectional study.
